# Rapid induction and long-term self-renewal of neural crest-derived ectodermal chondrogenic cells from hPSCs

**DOI:** 10.1038/s41536-022-00265-0

**Published:** 2022-12-08

**Authors:** Pei Shen, Lu Chen, Dahe Zhang, Simo Xia, Zhuman Lv, Duohong Zou, Zhiyuan Zhang, Chi Yang, Wenlin Li

**Affiliations:** 1 Department of Oral Surgery, Shanghai Ninth People’s Hospital, Shanghai Jiao Tong University School of Medicine; College of Stomatology, Shanghai Jiao Tong University; National Center for Stomatology; National Clinical Research Center for Oral Diseases; Shanghai Key Laboratory of Stomatology; Shanghai Research Institute of Stomatology; Research Unit of Oral and Maxillofacial Regenerative Medicine, Chinese Academy of Medical Sciences, Shanghai, China; 2 Department of Cell Biology, Naval Medical University, 200433 Shanghai, China

**Keywords:** Stem cells, Stem-cell biotechnology, Stem-cell research

## Abstract

Articular cartilage is highly specific and has limited capacity for regeneration if damaged. Human pluripotent stem cells (hPSCs) have the potential to generate any cell type in the body. Here, we report the dual-phase induction of ectodermal chondrogenic cells (ECCs) from hPSCs through the neural crest (NC). ECCs were able to self-renew long-term (over numerous passages) in a cocktail of growth factors and small molecules. The cells stably expressed cranial neural crest-derived mandibular condylar cartilage markers, such as MSX1, FOXC1 and FOXC2. Compared with chondroprogenitors from iPSCs via the paraxial mesoderm, ECCs had single-cell transcriptome profiles similar to condylar chondrocytes. After the removal of the cocktail sustaining self-renewal, the cells stopped proliferating and differentiated into a homogenous chondrocyte population. Remarkably, after transplantation, this cell lineage was able to form cartilage-like structures resembling mandibular condylar cartilage in vivo. This finding provides a framework to generate self-renewing cranial chondrogenic progenitors, which could be useful for developing cell-based therapy for cranial cartilage injury.

## Introduction

Articular cartilage that lines the synovial joints is formed by a distinct subpopulation of chondrocytes known as articular chondrocytes that are highly specified and persist throughout adult life. Articular cartilage is avascular with a very dense extracellular matrix (ECM), and articular chondrocytes have limited capacity for regeneration. Therefore, replacement therapies are the only means of treating patients with advanced articular injuries. Among these methods, chondrocyte and cartilage replacements are most commonly used^1^. Typically, articular chondrocytes are harvested from patients; however, the limited resources and complexity of the procedure have hindered wider application^2^. Although adult stem cells such as mesenchymal stem cells (MSCs) also have the potential to differentiate into chondrocytes, limitations in their proliferative ability and the heterogeneity of the cell population hinder their use as a prime alternative source for chondrocyte generation^3^.

To overcome these difficulties, embryonic stem cells (ESCs), which hold great promise for regenerative medicine, may provide an ideal alternative source of chondrocytes. To date, several approaches have been employed to harness the chondrogenic potential of ESCs for cartilage tissue engineering and regeneration^4,5^. Pioneering studies in ESCs involved differentiation via embryoid body (EB) formation, an in vitro system that to a certain extent mimics early embryonic development with the formation of three germ layers. However, the cartilaginous tissues formed via EBs were not homogenous, indicating the presence of other cell types in the pellets^6^. A high-density three-dimensional (3D) culture system for the chondrogenic differentiation of murine ESCs, which facilitates overall cell-to-cell and cell-to-matrix interactions and mimics in vivo limb development, provides a suitable environment for the chondrogenic differentiation of intact ESC-derived EBs^7^. Growth factors and cytokines can be used to establish a defined culture milieu for directing the chondrogenic differentiation of ESCs. The transforming growth factor-β (TGF-β) family, insulin-like growth factor (IGF) and sonic hedgehog (SHH) are the most potent inducers of chondrogenesis^8^. Yamashita et al. found that chondrogenic induction and cartilage formation in mouse ESCs were best induced using minimal serum conditions with TGF-β1 and bone morphogenetic protein 2 (BMP-2)^9^. Coculture with mature chondrocytes, limb bud progenitor cells, or mesodermal cells prior to induction has also been proposed to enhance the chondrogenic differentiation of human ESCs (hESCs)^10^. However, coculture systems may pose issues of contamination by the coculture cell type, thus affecting the yield and purity of the differentiated cells. In addition to growth factors, small molecules also play a vital role in stem cell chondrogenesis. Small molecules, such as dexamethasone, ascorbic acid and sodium pyruvate, have been widely used along with chondrogenic growth factor inducers in the chondrogenic differentiation of ESCs^11^.

Although there are numerous approaches for inducing ESC differentiation into chondrocytes, most of the methods involve the mesoderm^12–14^. During embryonic development, cartilage is derived from one of three precursor cell types: paraxial mesoderm, lateral plate mesoderm, and cranial neural crest (CNC). Limb buds are formed from paraxial and lateral plate mesoderm. Cartilage development in the limb rudiment starts from the cartilage anlage. A separate zone of chondrocytes at the ends of rudiments forms articular chondrocytes, which produce articular cartilage and remain throughout life^15^. However, the majority of craniofacial structures, including the temporomandibular joint (TMJ) condylar cartilage, are CNC-derived and are different from mesoderm-derived joints^16^. During embryonic development, the CNC migrates and populates the first pharyngeal arch to form paired mandibular processes that extend to form Meckel’s cartilage, which prefigures the mandible. A blastema forming the condylar cartilage develops beneath the periosteum from the ramal surface of the developing mandibular body^17^. Some studies have suggested that, although chondrocytes share similar phenotypic characteristics, including the synthesis of cartilage-specific proteoglycans, such as collagen II and collagen X, CNC-derived chondrocytes differ from those derived from the mesoderm. Ishizeki K et al. compared chondrocyte features between Meckel’s cartilage and costal cartilage, and they found that collagen I was detected only in cells from Meckel’s chondrocytes^18^. An increasing number of reports have confirmed that chondrocytes of different origins are characterized by different phenotypes, different ECMs, and different in vivo structures^19^.

Because of the origin and phenotypic differences, chondrocytes differentiated from hESCs via the mesoderm may not be suitable for repairing cartilage derived from the CNC, such as condylar cartilage. Although several studies have reported that the CNC can generate chondroprogenitors or chondrocytes, the presence of different cell types in the induced population is an obvious issue, and cell selection is usually needed to obtain a pure population^20,21^. In addition, chondrocytes derived from hESCs are highly specific and have no capacity to self-renew. The generation of self-renewing chondrogenic progenitors could be especially useful in therapeutic applications that require chondrocytes on a larger scale. In this study, we obtained CNC-derived self-renewing chondrogenic progenitors, which displayed uniform chondrocyte differentiation after the withdrawal of self-renewal conditions. Moreover, after transplantation, the cells formed TMJ condylar cartilage-like structures in vivo. Our study offers a renewable source of chondrogenic progenitors with the potential to treat joint injury and degeneration.

## Results

### Generation of self-renewing neural crest-derived progenitor cells expressing cartilaginous progenitor and stem cell markers from hPSCs

**Fig. 1:**
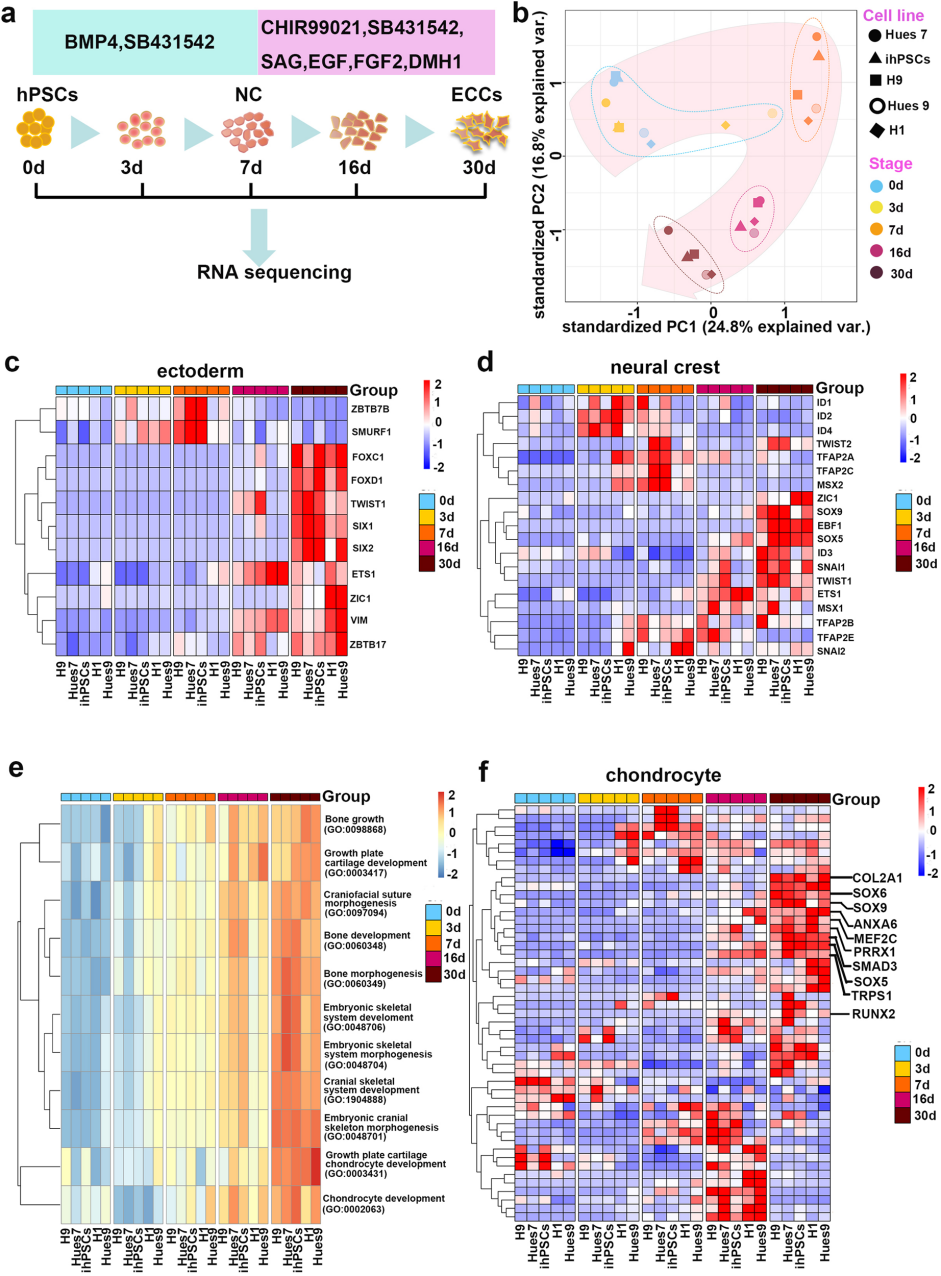
Generation of NC-derived progenitor cells from hPSCs. **a** Schematic representation of the NC-derived progenitor cell induction process. **b** PCA indicates that five unique hPSC (H1, Hues9, H9, Hues7 and ihPSC) lines followed similar differentiation trajectories. **c** Genes encoding ectoderm markers were upregulated during induction. **d** Genes encoding NC markers were upregulated during induction. **e** GO analysis highlighted bone and cartilage development, especially cranial skeleton morphogenesis and cranial skeletal system development, in NC-derived progenitor cells. **f** Genes encoding chondrogenesis markers were upregulated during induction.

Human pluripotent stem cells (hPSCs) were cultured on Matrigel-coated surfaces in an E8 medium. Neural crest (NC) was induced by switching from hPSC growth medium to NC induction medium consisting of DMEM/F12, N2, B27, 20 ng/ml BMP4 and 2 μM the TGF-β receptor inhibitor SB431542 for 7 days. Previously, we successfully self-renewed primitive neural stem cells derived from hESCs by using a medium containing the glycogen synthase kinase-3 (GSK-3) inhibitor CHIR99021, SB431542, and human leukaemia inhibitory factor^22^. In attempting to induce the self-renewal of this BMP4- and SB431542-induced NC population, we fortuitously defined a culture medium formulation that could stably maintain the self-renewal of this NC population. The medium consisted of DMEM/F12 supplemented with N2, B27, 1 μM CHIR99021, 2 μM SB431542, 0.1 μM smoothened agonist (SAG), 20 ng/ml epidermal growth factor (EGF), 20 ng/ml fibroblast growth factor 2 (FGF2), and 1 μM BMP receptor inhibitor (DMH1), and is hereafter referred to as CSSEDF (Fig. 1a). RNA-seq was performed for five hPSC cell lines, including H1, Hues9, H9, Hues7 and ihPSC (ATCC-DYR0100), on Days 3, 7, 16 and 30 after induction. Principal component analysis (PCA) suggested that the induction protocol was highly reproducible across different hPSC lines (Fig. 1b). Immunostaining showed that NC markers, such as AP2, DLX2, and ETS1^23^, were uniformly expressed in hPSCs treated with BMP4 and SB431542 for 7 days (Supplementary Fig. 1a), suggesting the induction of NC cells. RNA-seq analysis at different induction points showed that markers representing neural ectoderm and NC were upregulated sequentially during the induction process (Fig. 1c, d)^24^. Real-time PCR analysis revealed a rapid loss of OCT4, NANOG, and SOX2 expression after treatment with BMP4 and SB431542 for 7 days (Supplementary Fig. 1b). However, the expression of neural ectodermal and neural plate border markers, SIX1, TUBB3 and ZIC1^25–27^, was upregulated after induction. In addition, the expression of the mesodermal markers KDR, TBX6, and PAX1^28–30^ and the endodermal markers GATA4, GATA6, and SOX7^31,32^ were not significantly induced after treatment with CSSEDF medium (Supplementary Fig. 1b). All these data suggest the successful generation of NC populations from hPSCs after 7 days of treatment with BMP4 and SB431542.

After treatment with CSSEDF medium, the cell morphology gradually changed to mesenchymal monolayers (Supplementary Fig. 1c, d). To determine the nature of CSSEDF-expanded cells, gene ontology (GO) enrichment analysis of the genes using the R package GAGE was performed. Significantly upregulated GO terms in biological processes highlighted bone and cartilage development, especially cranial skeleton morphogenesis and cranial skeletal system development (Fig. 1e). We next compared the TCGA transcriptome data between cells at different induction stages to determine the differentially expressed genes. The top 10 upregulated and downregulated genes are listed in Supplementary Fig. 2a. Consistently, gene set enrichment analysis (GSEA) showed a significant upregulation of the cranial skeleton and cartilage gene set during induction (Supplementary Fig. 2b). Chondrogenic markers such as SOX5, SOX6, SOX9, RUNX2, COL2A1, ANXA6, MEF2C, PRRX1, SMAD3, and TRPS1 were upregulated after induction for 30 days (Fig. 1f)^14,33–37^. PCR analysis revealed that the expression of the chondrogenic genes SOX9, ACAN, RUNX2, SP1, and OCN was dramatically upregulated during the induction process (Supplementary Fig. 2c). Taken together, these data suggest that our dual-phase induction may produce an ectodermal chondrogenic lineage.

**Fig. 2:**
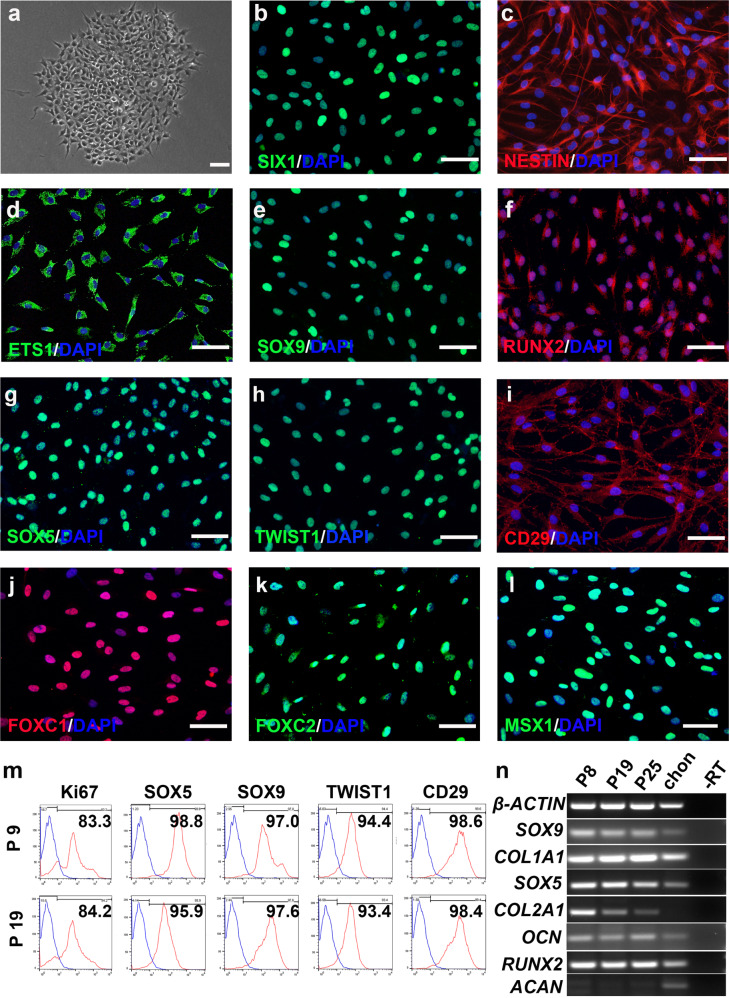
CSSEDF-expanded cells stably self-renewed and shared markers of CPCs/CSCs. **a** A single CSSEDF-expanded cell (passage 5) colony on a Matrigel-coated surface. Scale bars, 50 µm. **b**–**d** Immunocytochemistry showing that CSSEDF-expanded cells expressed genes identified as neural ectodermal markers, including SIX1, NESTIN and ETS1. Scale bars, 50 µm. **e–i** Immunocytochemistry showing that CSSEDF-expanded cells (passage 5) expressed genes identified as CPC/CSC markers, including SOX9, RUNX2, SOX5, TWIST1, and CD29. Scale bars, 50 µm. **j–l** Immunocytochemistry showing that CSSEDF-expanded cells (passage 5) express genes recently identified as TMJ condylar cartilage markers, including FOXC1, FOXC2 and MSX1. Scale bars, 50 µm. **m** Flow cytometry analysis showing that CSSEDF-expanded cells stably express cell proliferation and CPC/CSC markers after long-term in vitro expansion. **n** Gene expression of chondrocytic lineage markers by CSSEDF-expanded cells at different passages and patient TMJ condylar cartilage were analysed by PCR. CPCs/CSCs: cartilaginous progenitor cells/cartilaginous stem cells.

CSSEDF can sustain the long-term self-renewal of these cells on Matrigel-coated surfaces. CSSEDF-expanded cells were routinely passaged at 1:10 and cultured for >30 passages without obvious loss of proliferative capacity. Single cells at passage 5 and passage 16 were clonogenic on Matrigel in the presence of CSSEDF (Fig. 2a and Supplementary Fig. 3a). Immunostaining showed that neural ectodermal markers, including SIX1, NESTIN and ETS1^26,38^, were positive in the CSSEDF-expanded cells (passage 5; Fig. 2b–d), which is consistent with their NC origin. Immunostaining revealed that both early-passage (passage 5) and late-passage (passage 18) CSSEDF-expanded cells stably expressed genes identified as cartilaginous progenitor/stem cell (CPC/CSC) markers, including SOX9, RUNX2, SOX5, TWIST1, and CD29 (Fig. 2e–i and Supplementary Fig. 3b-f)^39^. SOX9 is a universally accepted marker of chondrogenesis. However, SOX9 does not work alone, and its function depends on the coexpression of SOX5/6^40^. RUNX2 is a master regulator of chondrocyte specification and differentiation^39^. TWIST1 can inhibit the differentiation of CPCs/CSCs into downstream cell types and maintain the characteristics of CPCs/CSCs^41^. CD29 is reported to be most highly expressed in CPCs and plays an important role in the chondrogenic differentiation and cartilage tissue formation of CPCs^42^. These results suggest that CSSEDF-expanded cells share CPC/CSC markers. Notably, immunostaining confirmed the expression of genes recently identified as neural crest-derived TMJ condylar cartilage markers^43^, such as FOXC1, FOXC2 and MSX1, in CSSEDF-expanded cells (Fig. 2j–l). The stable phenotype of CSSEDF-expanded cells after extensive passages was further confirmed by flow cytometry. Both early-passage (passage 9) and late-passage (passage 19) CSSEDF-expanded cells exhibited nearly identical expression patterns for a set of chondrocyte-specific markers, such as SOX5 (98.8% and 95.9% positive), SOX9 (97.0% and 97.6% positive), TWIST1 (94.4% and 93.4% positive), and CD29 (98.6% and 98.4% positive) and the cell proliferation marker Ki-67 (83.3% and 84.2% positive; Fig. 2m). PCR further demonstrated that early-passage (passage 8) and late-passage (passages 19 and 25) CSSEDF-expanded cells stably expressed genes identified as chondrogenic markers, including SOX9, COL1A1, SOX5, COL2A1, OCN and RUNX2 (Fig. 2n).

### CSSEDF-expanded cells have a propensity to differentiate into chondrocytes

Next, we evaluated the spontaneous differentiation of CSSEDF-expanded cells in N2B27 basal medium free of CSSEDF self-renewal supplements in the absence of any inducing agents. CSSEDF-expanded cells were evaluated in monolayer, collagen sponge and suspension culture conditions. Under monolayer conditions, the cells stopped proliferating. Scattered white flakes were visible in the Petri dish. Haematoxylin and eosin (HE) staining of these flakes revealed that cartilage lacuna was formed and that the ECM was rich after 8 weeks in monolayer culture (Supplementary Fig. 4). We further evaluated the expression of osteogenic differentiation markers ALPL, SPP1, and SP7, and adipogenic differentiation markers FABP4, CEBPA and APN in CSSEDF-expanded cells on Day 0 and Week 8 after spontaneous differentiation under monolayer condition. RT-PCR analysis showed that the expression of osteogenic and adipogenic differentiation markers was virtually undetectable (Supplementary Fig. 5). These data confirmed that CSSEDF-expanded cells were specifically chondrogenic, and these cells are hereafter referred to as ectodermal chondrogenic cells (ECCs).

**Fig. 3:**
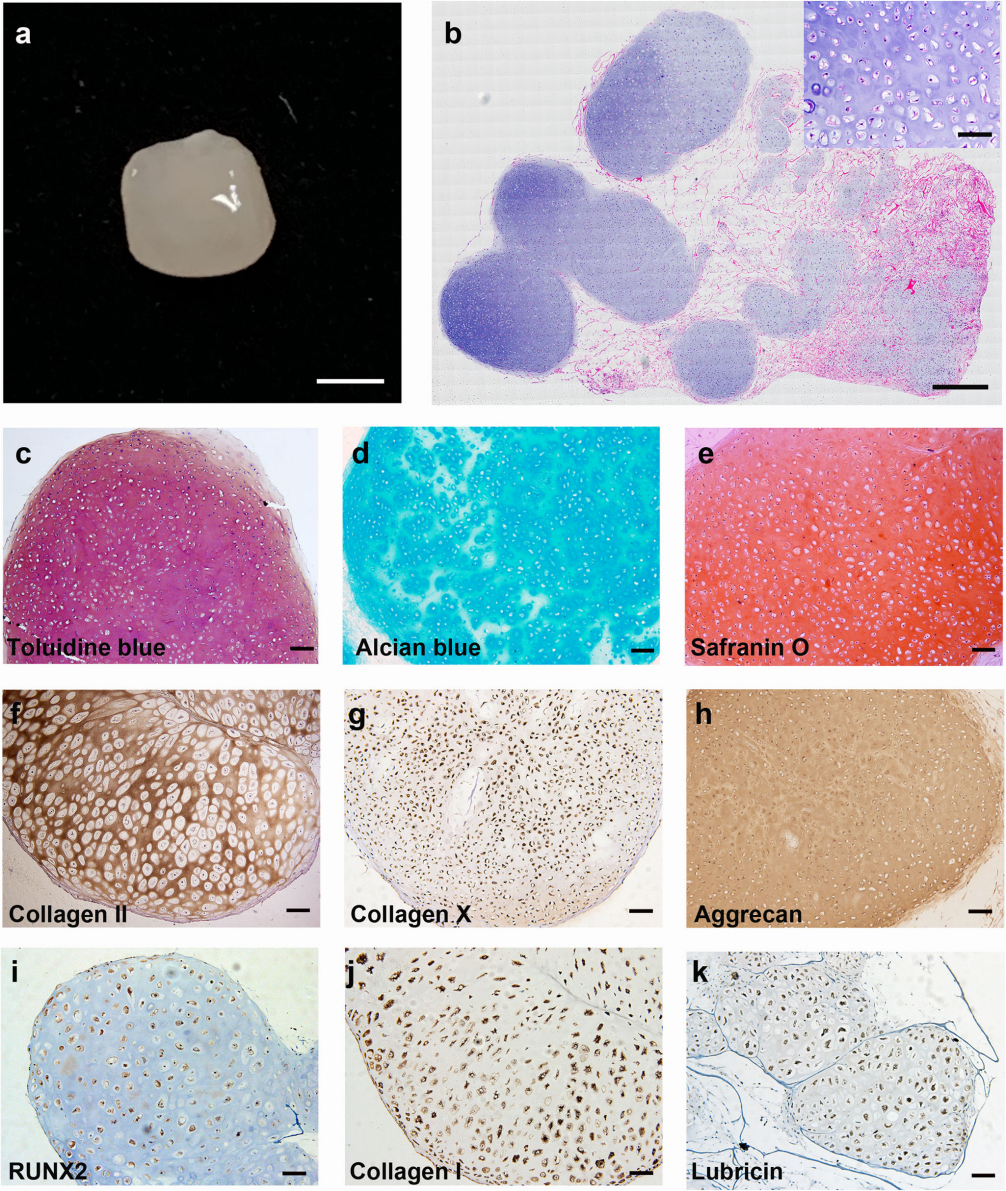
ECCs can spontaneously differentiate into chondrocytes in vitro. **a** Gross appearance of ECCs seeded in a collagen sponge for 8 weeks. Scale bars, 1 mm. **b** Panoramic scanning showing the formation of cartilage masses in the collagen scaffold (inset: HE staining enlargement). Scale bars, 200 µm. (Inset) Scale bars, 50 µm. **c**–**e** The chondrocytes were positive for toluidine blue, alcian blue and safranin O staining. Scale bars, 100 µm. **f**–**k** Immunohistochemical staining showing that the chondrocytes express common chondrocytic markers, including collagen II, collagen X, aggrecan, RUNX2, collagen I, and the articular cartilage marker lubricin. Scale bars, 100 µm.

ECCs were seeded onto a collagen sponge and cultured with N2B27 medium. After 4-week culture, the cells in the sponge were largely immature chondrocytes (Supplementary Fig. 6). At 8 weeks, the collagen sponge developed a smooth and glistening appearance (Fig. 3a). Panoramic scanning of HE staining showed scattered cartilage masses in the collagen scaffold. Chondrocytes were large and buried in the cartilage lacuna with rich ECM (Fig. 3b) and strong positive toluidine blue, alcian blue and safranin O staining, indicative of proteoglycan-rich cartilage-like ECM (Fig. 3c–e). The cartilage masses expressed collagen II, collagen X, aggrecan and RUNX2, as expected (Fig. 3f-i). Moreover, collagen I was also detected, consistent with its known expression in mandibular condylar chondrocytes (Fig. 3j). Interestingly, lubricin, a heavily O-glycosylated protein^44^ that is essential for boundary lubrication of articular cartilage, was also detected (Fig. 3k). ECCs were also cultured in suspension using ultralow attachment plates. The cells formed spheres in CSSEDF medium after 5 days. The medium was then changed to N2B27, and the growth of spheres slowed significantly, with spheres adopting a glistening appearance (Supplementary Fig. 7a). Panoramic scanning showed homogenous chondrocytes with rich ECM in the sphere, but the cells and cartilage lacuna were smaller than the chondrocytes differentiated in the collagen sponge (Supplementary Fig. 7b). Sphere tissues showed strong toluidine blue and alcian blue staining, while safranin O staining was weak. The sphere tissue also demonstrated strong expression of collagen II, collagen X and collagen I (Supplementary Fig. 7c–h).

**Fig. 4:**
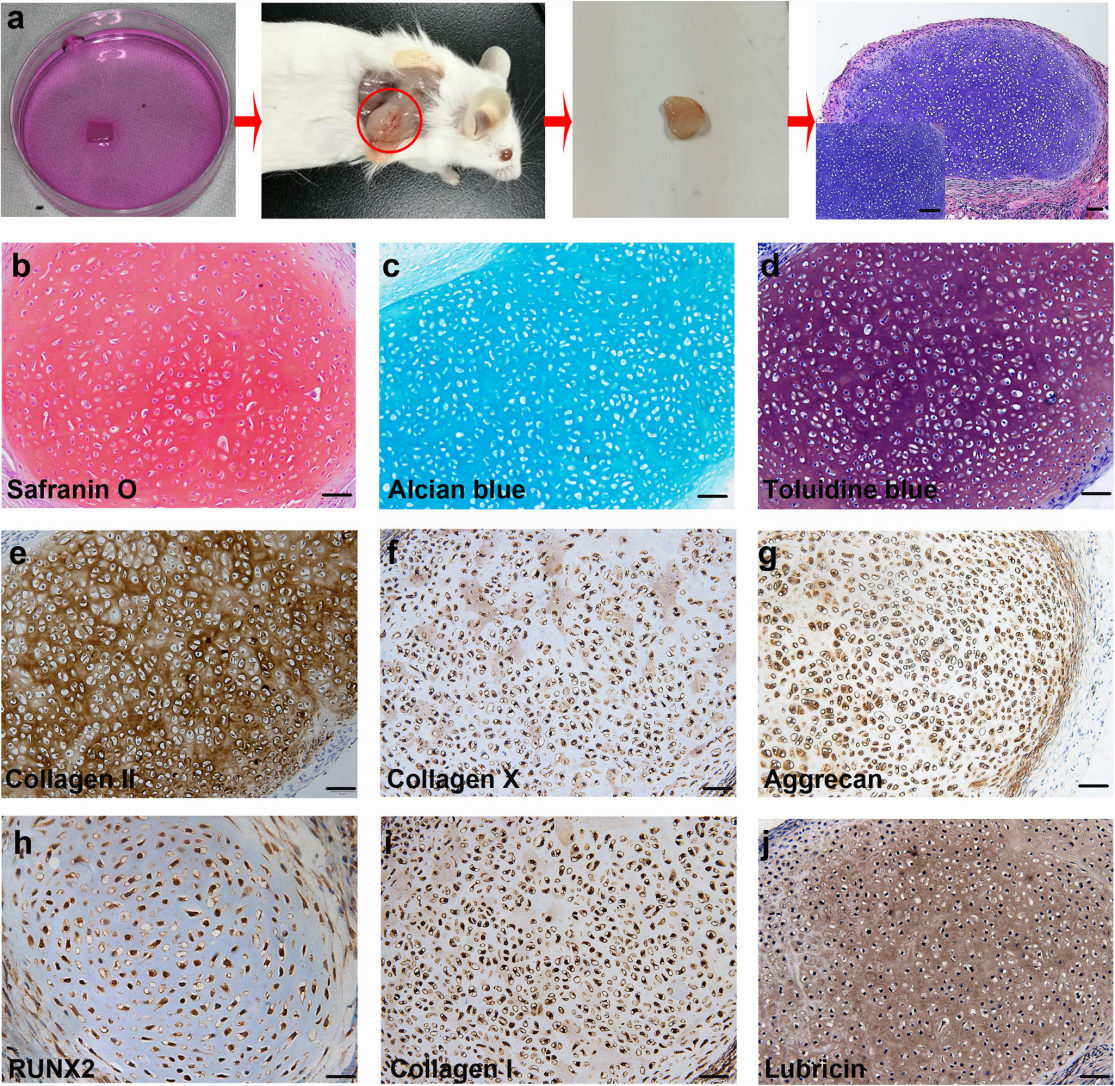
ECCs can generate cartilage in vivo without teratoma formation. **a** Schematic representation showing ECCs seeded in a collagen sponge in vitro for 4 weeks and surgically transplanted subcutaneously into the dorsum of NOD-SCID mice. The implants were harvested after 4 weeks and histologically confirmed to be cartilage. (Inset) Enlargement of HE staining. Scale bars, 200 µm. (Inset) Scale bars, 50 µm. **b–d** Harvested cartilage was positive for safranin O, alcian blue and toluidine blue staining. Scale bars, 100 µm. **e**–**j** Immunohistochemical staining showing that differentiated chondrocytes express common chondrocytic markers, including collagen II, collagen X, aggrecan, RUNX2, collagen I, and the articular cartilage marker lubricin. Scale bars, 100 µm.

To further characterize the differentiation potential of hPSC-derived ECCs, a collagen sponge seeded with ECCs and cultured for 4 weeks was transplanted subcutaneously into the dorsum of NOD-SCID mice. Histological analysis confirmed no teratoma formation in the transplanted sponge at 4 weeks (Fig. 4a). Strong positive safranin O, alcian blue and toluidine blue staining in the sponge indicated proteoglycan-rich cartilage-like ECM (Fig. 4b–d). The generated cartilage tissue expressed collagen II, collagen X, aggrecan and RUNX2 (Fig. 4e–h). Collagen I and lubricin were also detected in the grafts, which was similar to the staining pattern found in the tissue in vitro (Fig. 4i, j). Together, these results demonstrate that ECCs can spontaneously differentiate into chondrocytes both in vitro and in vivo after the withdrawal of self-renewal factors.

### ECCs have similar single-cell transcriptome profiles to TMJ condylar chondrocytes

**Fig. 5:**
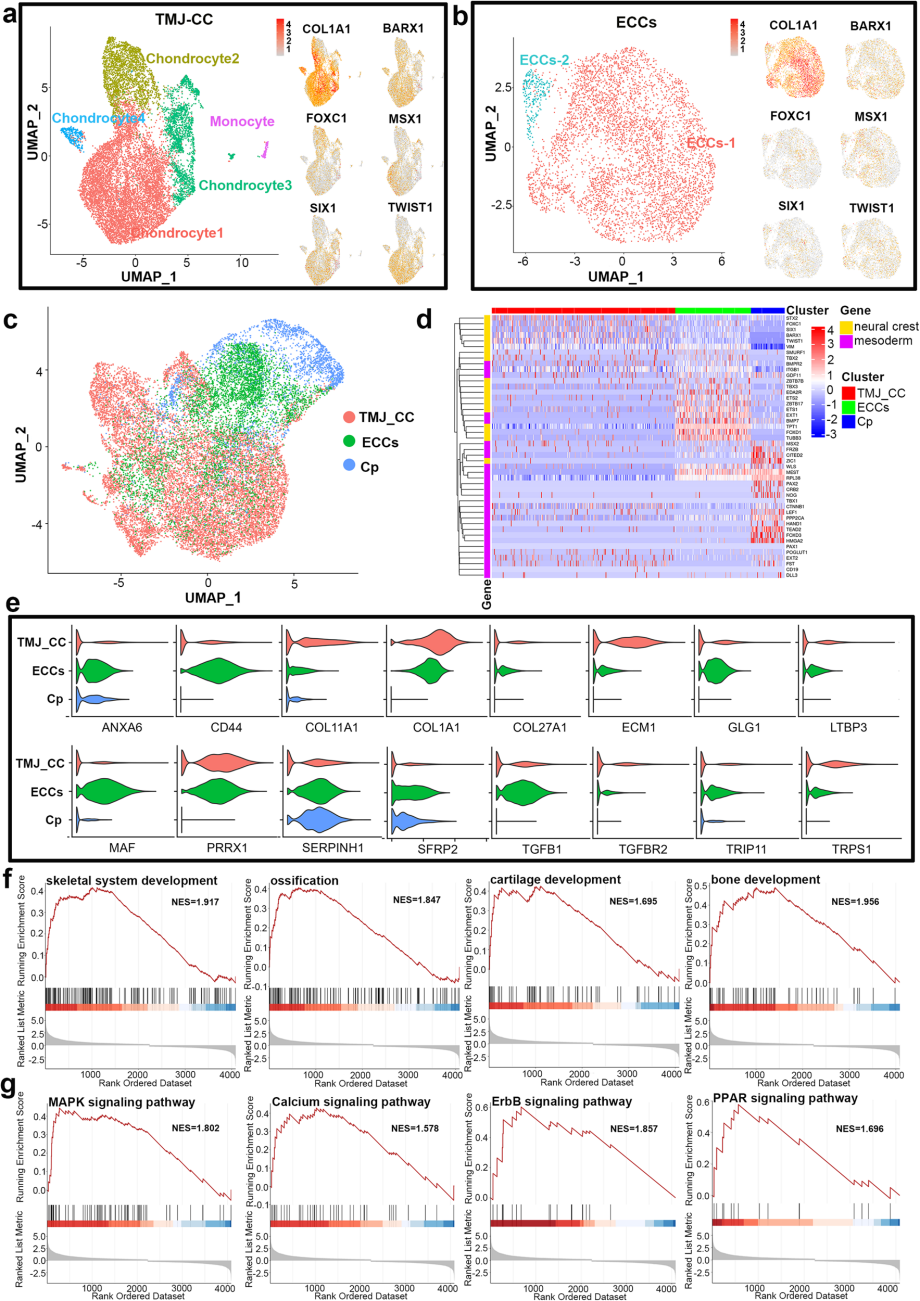
ECCs have similar single-cell transcriptome profiles to TMJ condylar chondrocytes. **a** and **b** Both TMJ-CC and ECCs were enriched in mandibular condyle and neural crest markers, such as COL1A1, BARX1, FOXC1, MSX1, SIX1, and TWIST1. **c** UMAP confirmed that ECCs are closer to TMJ-CC than Cp from hiPSCs via the paraxial mesoderm. **d** Both the TMJ-CC and ECCs exhibited high expression of neural crest markers, while genes involved in mesoderm were clustered in the Cp. **e** Violin plots showing that TMJ-CC and ECCs presented nearly the same chondrogenic markers. **f** GSEA suggesting that ECCs had different gene expression from Cp in skeletal system development, ossification, cartilage development, and bone development. **g** MAPK, calcium, ErbB, and PPAR signalling pathway-associated genes were different between ECCs and Cp.

The neural crest contributes to early craniofacial development by generating mesenchymal tissues, including chondrocytes. As noted, ECCs expressed the genes recently identified as neural crest-derived TMJ condylar cartilage markers, such as FOXC1, FOXC2 and MSX1. Next, single-cell transcriptome profiles of TMJ condylar chondrocytes (TMJ-CC) and ECCs were acquired. Identification of subpopulations showed that TMJ-CC were comprised of four clusters of chondrocytes (chondrocyte 1, *n* = 7688, 62.1%; chondrocyte 2, *n* = 2205, 17.8%; chondrocyte 3, *n* = 2074, 16.7%; and chondrocyte 4, *n* = 304, 2.4%) and a small cluster of monocytes (*n* = 115, 0.9% of the total population; Fig. 5a). Both TMJ-CC and ECCs were enriched in the mandibular condyle and NC markers, such as COL1A1, BARX1, FOXC1, MSX1, SIX1, and TWIST1 (Fig. 5a, b), indicating that TMJ-CC and ECCs exhibited similar developmental origins.

Next, we compared ECCs, TMJ-CC and chondroprogenitor (Cp, a lineage from hiPSCs via the paraxial mesoderm)^45^ based on single-cell data. Single-cell RNA-seq data were subjected to unsupervised clustering and visualized using uniform manifold approximation and projection dimension reduction analysis (UMAP). We observed that ECCs are closer to TMJ-CC than Cp (Fig. 5c). Notably, both the TMJ-CC and ECCs exhibited high expression of NC markers, while genes involved in mesoderm were clustered in the Cp (Fig. 5d), which was in accordance with their trajectories. Compared with Cp, violin plots showed that TMJ-CC and ECCs presented nearly the same chondrogenic markers (Fig. 5e). Although they were both chondroprogenitors, GSEA suggested that ECCs had different gene expression from Cp on skeletal system development, ossification, cartilage development, and bone development (Fig. 5f). We also observed the distribution and enrichment score of MAPK, calcium, ErbB, and PPAR signalling pathway-associated genes between the ECCs and Cp (Fig. 5g). The top 10 significantly differentially expressed (SDE) genes of TMJ-CC, ECCs and Cp were further represented in the heatmap shown in Supplementary Fig. 8a. GO analysis of these dynamically expressed genes indicated that cranial suture morphogenesis, craniofacial suture morphogenesis, and cartilage development involved in endochondral bone morphogenesis were enriched in both ECCs and TMJ-CC (Supplementary Fig. 8b). Kyoto Encyclopaedia of Genes and Genomes (KEGG) analyses of these dynamically expressed genes indicated that neurotrophin, TGF-beta, calcium, insulin, and JAK-STAT signalling pathways were remarkably enriched in major clusters of TMJ-CC and ECCs (Supplementary Fig. 8c).

To further evaluate the profile of ECCs, we compared TMJ-CC with the cells differentiated for 28 days from ECCs and Cp by single-cell transcriptome analysis. Interestingly, a cluster of cells in ECC-28d overlapped with TMJ-CC, while only a few cells in Cp-28d were close to TMJ-CC (Supplementary Fig. 9a). Further clustering analyses yielded 7 major clusters of cell types (Supplementary Fig. 9b). Clusters 1–4 were chondrocytes, and Clusters 5, 6 corresponded to progenitor cells. Cluster 7 was monocytes from TMJ-CC. Both ECC-28d and TMJ-CC expressed fibrocartilaginous markers (e.g., COL3A1 and COL1A2), cranial chondrocyte markers (e.g., GNAS, BARX1, MGP)^46–48^, and craniofacial markers (e.g., DCN, ITGB1)^49,50^. Cp-28d mainly expressed hypertrophic chondrocyte markers in the growth plate (e.g., CYR61, CXCR4, CTGF)^51,52^, primary chondrocyte markers (e.g., GPX1)^53^ and mesoderm markers (e.g., MESDC2)^54^ (Supplementary Fig. 9c).

### hPSC-derived ECCs can be used to repair cartilage defects

**Fig. 6:**
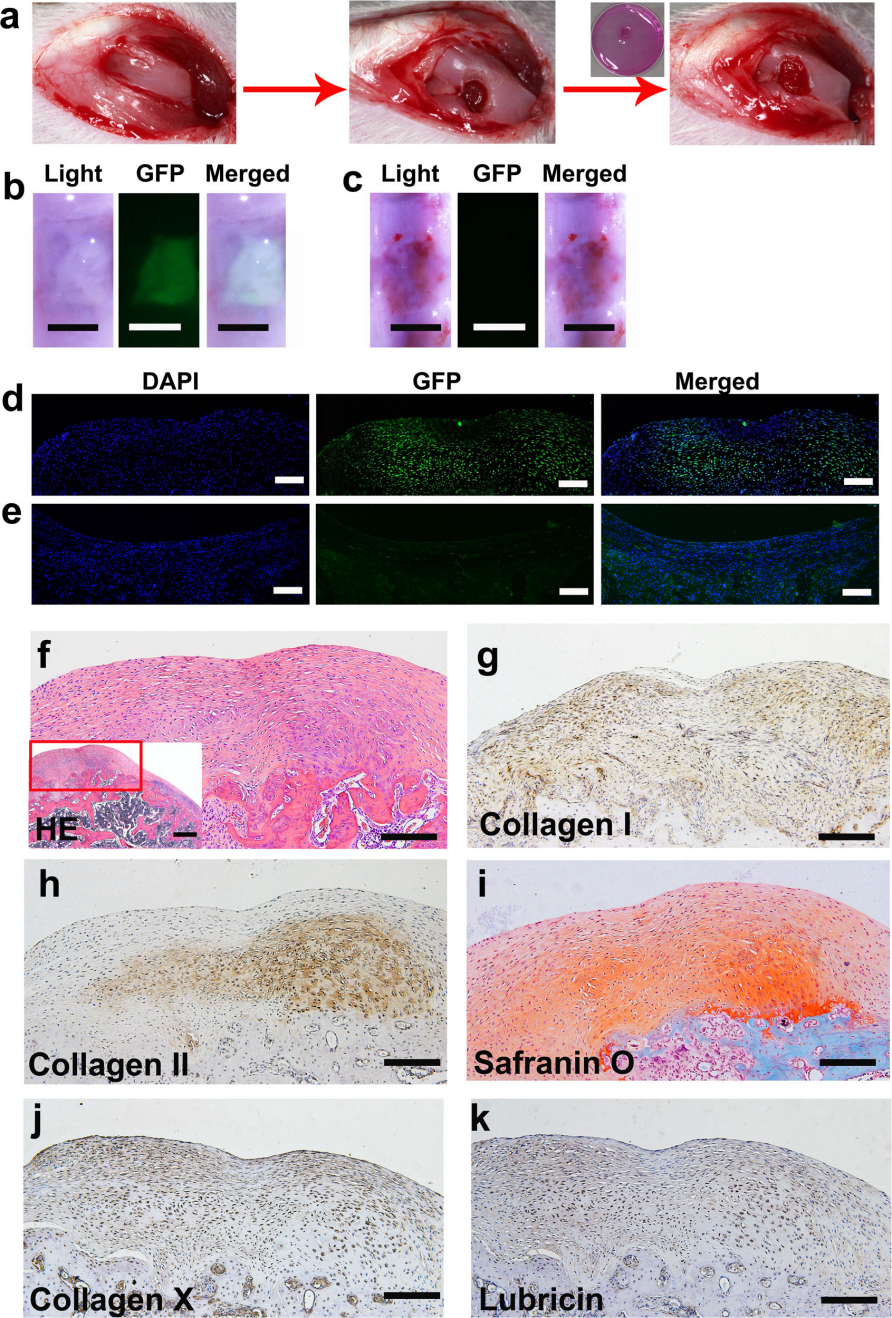
ECCs can repair knee defects and form layered cartilage structures. **a** Schematic representation showing that a full-thickness rat cartilage defect was created. ECCs were seeded in collagen sponges, cultured in vitro for 4 weeks, and then surgically transplanted into cartilage defects. **b**, **d** Knees treated with GFP-expressing ECCs loaded on a collagen sponge were harvested 4 weeks after transplantation. GFP expression was detected in the ECC group. **b** Scale bar, 1 mm. **d** Scale bar, 50 μm. **c**, **e** No GFP expression was observed in the control group transplanted with only the collagen sponge. **c** Scale bar, 1 mm. **e** Scale bar, 50 μm. **f** HE staining showing that the repaired tissue was cartilage-like. (Inset) HE staining at low magnification. Scale bar, 100 μm. (Inset) Scale bar, 200 μm. **g** Immunohistochemical staining revealing that collagen I was present throughout the cartilaginous cell layers. **h** Immunohistochemical staining showing that collagen II was predominant in the deep layer. **i** Safranin O is more significantly positive than the deeper layer. **j**, **k** Immunohistochemical staining showing that collagen X and lubricin are expressed in the regenerated cartilage tissue. **g–k** Scale bar, 100 μm.

Cartilage defects are common disorders that mainly affect articular cartilage. hPSC-derived ECCs provide an opportunity to repair defective TMJ condylar cartilage derived from the CNC. However, it is difficult to establish a TMJ cartilage repair model in rats. Thus, we used a rat knee cartilage defect model to test the ability of hPSC-derived ECCs to repair articular cartilage. A full-thickness rat cartilage defect was created in each knee of rats. A collagen sponge with or without ECCs was implanted into the right or left knee defect, respectively (Fig. 6a). In the ECC group, the regenerated tissue fully filled the defect, smoothly integrated with the host cartilage, and presented a white glistening appearance. Green fluorescence was observed in the regenerated tissue, and the fluorescence exactly matched the repaired tissue under the fluorescence stereoscope (Fig. 6b). In the control group, damaged cartilage exhibited scarring with apparent poor regeneration. No green fluorescence was detected in the harvested tissue (Fig. 6c). Immunofluorescent staining showed that green fluorescent protein (GFP) expression was positive in the transplanted site 4 weeks later (Fig. 6d). Accordingly, no GFP was detected in the control group (Fig. 6e).

Mature TMJ condylar cartilage consists of the following four zones (Supplementary Fig. 10a)^16^, which are different from knee cartilage (Supplementary Fig. 10b): (1) articular surface of fibrous tissue expressing collagen I, (2) proliferative cells in the prechondroblastic zone expressing collagen I, (3) a chondroblastic zone expressing collagen II, proteoglycans aggrecan, decorin, chondroitin sulfate PG, and keratin sulfate PG, and (4) a hypertrophic zone adjacent to bone expressing collagen X. On the other hand, knee cartilage, as typical hyaline cartilage, demonstrates homogenous ECM, which is dominated by collagen II. Interestingly, in the ECC group, the regenerated tissue became part of a cartilage-like TMJ condyle with an obvious layered structure (Fig. 6f). The ECCs formed cartilage, as evidenced by collagen I expression throughout the cartilaginous cell layers, whereas collagen II and safranin-O staining for chondroitin sulfate PG were observed predominantly in the deep layer. Collagen X was detected both in the super and deep layers. Lubricin was positive in the regenerated cartilage tissue (Fig. 6g–k). In the control group, the scar tissue was thinner and consisted of fibrocytes with vascular invasion. Safranin-O staining and collagen I and collagen II immunofluorescence costaining showed the absence of chondrocyte ECM (Supplementary Fig. 11).

The above data demonstrate that hPSC-derived ECCs can generate articular cartilage, which is similar to TMJ condylar cartilage in the microenvironment of the articular cavity.

### Activation of the Wnt signalling pathway affects chondrogenic differentiation of ECCs

**Fig. 7:**
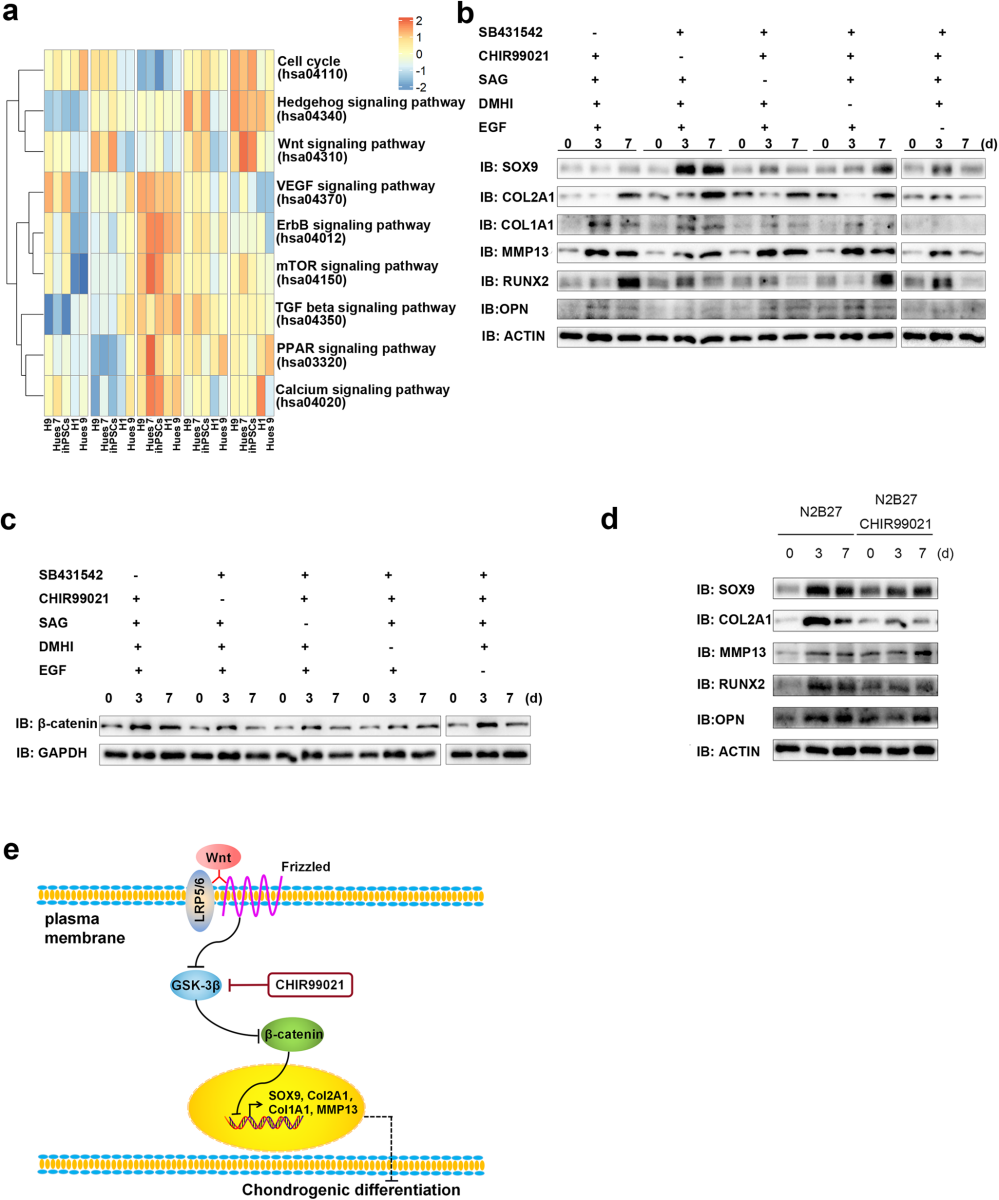
The activated Wnt signalling pathway affects chondrogenic differentiation of ECCs. **a** KEGG analysis of bulk RNA-seq at different induction points. **b** The transcription factor SOX9, condylar chondrocyte anabolism markers COL2A1 and COL1A1, and the catabolism marker MMP13 were obviously upregulated 3 days after the removal of CHIR99021. **c** An obvious downregulation of β-catenin was detected by western blotting 7 days after the removal of CHIR99021. **d** The chondrogenic transcription factors SOX9 and RUNX2 were decreased, as well as COL2A1 and OPN, while MMP13 was enhanced in the presence of CHIR99021 in N2B27. **e** A scheme for the Wnt signalling pathway involved in chondrogenic differentiation of ECCs.

KEGG analyses of bulk RNA-seq at different induction points showed that the hedgehog and Wnt signalling pathways were remarkably enriched at the last induction point (Fig. 7a). Considering the effects of the small molecules added to the CSSEDF culture medium, we reduced the small molecules one by one to evaluate the chondrogenic differentiation of ECCs. Western blotting analysis showed that the transcription factor SOX9, the condylar chondrocyte anabolism markers COL2A1 and COL1A1, and the catabolism marker MMP13 were obviously upregulated 3 days after the removal of CHIR99021 (Fig. 7b). CHIR99021, as a canonical Wnt signalling agonist, can activate β-catenin, a downstream Wnt mediator^55^. Obvious downregulation of β-catenin was detected 7 days after the removal of CHIR99021 by western blotting (Fig. 7c). To further confirm the role of Wnt signalling in chondrogenic differentiation, we compared the spontaneous differentiation of ECCs in N2B27 with or without CHIR99021. After adding the Wnt signalling agonist CHIR99021, the chondrogenic transcription factors SOX9 and RUNX2 were decreased, as well as COL2A1 and OPN, while MMP13 was enhanced (Fig. 7d). These results suggest that the activated Wnt signalling pathway can inhibit chondrogenic differentiation of ECCs (Fig. 7e).

## Discussion

Currently, it is tremendously challenging to obtain TMJ condylar cartilage tissue for research and therapy purposes. The isolation of primary condylar chondrocytes is highly invasive. As primary chondrocytes survive for a relatively short period of time and support limited generations, repeated sampling is usually needed for research. In particular, it is virtually impossible to harvest primary condylar chondrocytes in large quantities, which renders primary chondrocytes unsuitable for therapeutic transplantation. In addition, MSCs, represented by bone marrow stem cells (BMSCs), and chondroma cell lines, such as SW1353, are commonly used as surrogates for TMJ condylar chondrocytes. However, the chondrocyte differentiation of BMSCs is inefficient and difficult to control^56^. Chondroma cell lines are different from normal articular cartilage, especially mandibular cartilage chondrocytes, which are derived from the CNC. Consequently, there is an urgent need to develop renewable cell sources for TMJ condylar chondrocytes for research and therapy applications.

hPSCs can self-renew indefinitely and have the potential to generate any cell type in the adult body. Due to the significant challenges in the isolation and long-term expansion of most tissue-specific stem/progenitor cells from adults, the differentiation of hPSCs into renewable tissue-specific cell types is highly desirable for various biomedical applications. However, despite significant advances in the development of various chondrocyte induction conditions for hPSCs, most differentiation protocols use poorly defined culture conditions and eventually generate terminally differentiated chondrocytes^5,13^. In addition, most induction conditions usually yield mixed populations or even other embryonic germ layer lineages^6^. The development of self-renewing chondrogenic progenitors from hPSCs is highly desired. In the present study, our observations led us to develop robust chemically defined conditions using specific small molecules that rapidly and uniformly convert hPSCs into ECCs. Consistent with TMJ condylar cartilage development, our induction process was confirmed to occur through the CNC. Well-acknowledged markers of the development of the CNC, such as DLX2, AP-2 and ETS1^24^, were highly expressed during the induction process. NESTIN, an NC stem cell marker^57^, and SIX 1, a crucial transcription factor in facial bone and cartilage development^58^, were uniformly expressed in ECCs, as observed by immunofluorescent staining. Above all, the present method enables the long-term expansion of ECCs without loss of the ability to self-renew. ECCs possess features of chondrogenic precursor cells and can spontaneously differentiate into chondrocytes with high efficiency in the absence of self-renewing factors in defined tissue culture media.

To our knowledge, this is the fastest and most efficient method to produce cartilaginous progenitors from hPSCs through the CNC. Notably, ECCs expressed critical genes participating in facial patterning mandibular chondrocyte development, including DLX2, MSX1, FOXC1 and FOXC2. A previous study showed altered expression of several facial patterning genes, including DLX2, MSX1, FOXC1 and FOXC2, indicating mispatterned CNC-derived cells in the facial region^59^. MSX1 is upregulated during NC differentiation and can promote chondrocyte-like cell formation^60^. In NC-derived cells, Fox proteins are sufficient for cartilage development. FOXC1-null embryos display bony syngnathia together with defects in maxillary and mandibular structures and agenesis of the TMJ^43^. FOXC2-mutant mice present defective TMJ condylar cartilage development^61^. Single-cell RNA-seq further confirmed that ECCs share similar profiles to mandibular condylar chondrocytes compared with Cp originating from hiPSCs via the paraxial mesoderm. In the critical-sized rat knee cartilage defect repair model, collagen sponges seeded with ECCs achieved dramatic cartilage repair and integration and formed a structure similar to that of TMJ condylar cartilage. These results indicated that ECCs differ from previously reported hESC-derived CPCs/CSCs in that they adopt a chondrocyte phenotype that is close to that of mandibular chondrocytes both in vitro and in vivo. Considering the significance of developing renewable sources of ectodermal chondrocytes, it will be useful to study the physiological and pathological mechanisms of mandibular condyles and evaluate the effect of ECCs on mandibular condylar disorders. Consequently, this protocol also provides a valuable tool to study the early molecular events initiating human mandibular chondrocyte induction.

## Methods

### Cells and culture conditions

hESCs, H1 cells (passages 40–50), Hues9 cells (passages 17–30), H9 cells (passages 40–50), Hues7 cells (passages 15–20) and iPSCs (ATCC-DYR0100, passages 15–20) were cultured in E8 medium on 2% Matrigel (growth factor reduced, BD Biosciences)-coated dishes. hPSCs were passaged using Accutase (Millipore) at a dilution of 1:10. Blebbistatin (Selleck, 5 μM) was used to improve cell attachment after passaging but was not used in the routine culture of hPSCs. Human preadipocytes (BFN608007090, Shanghai Cell Bank, passages 15–20), human osteoblasts (hFOB1.19, Shanghai Cell Bank, passages 18-22) were cultured in DMEM high glucose + 10% FBS + 1% Penicillin/Streptomycin (Gibco), and both were passaged using Trypsin (Gibco) at a dilution of 1:2.

### ECC Induction

hPSCs (H1, Hues9, H9, Hues7 and iPSCs) at approximately 20% confluence were treated with 20 ng/ml BMP (PeproTech) and 2 μM SB431542 (Selleck) in DMEM/F12 medium supplemented with 1× N2, 1× B27 and 1% penicillin/streptomycin, hereafter referred to as N2B27 medium, for 7 days. The culture was then split 1:6 using Accutase and cultured in N2B27 medium supplemented with 1 μM CHIR99021, 2 μM SB431542, 0.1 μM SAG, 20 ng/ml EGF, 20 ng/ml FGF2, and 1 μM DMH1, hereafter referred to as CSSEDF medium, on Matrigel-coated plates. These hPSC-derived ECCs were maintained stably in vitro.

### Spontaneous differentiation

To detect the potential of spontaneous differentiation, ECCs at passage 10 were cultured in monolayers (1 × 10^5^ cells/cm^2^) for 8 weeks and in suspension (Corning ultralow attachment culture dishes; 1 × 10^6^ cells/cm^2^) for 8 weeks or seeded in a collagen sponge (12 × 12 × 3 mm; Helistat 1690ZZ; 1 × 10^7^ cells/cm^2^) for 8 weeks. To seed ECCs into a collagen sponge, ECC suspension was dropped into the sponge and placed in a corning ultralow attachment dish without a medium. The sponge soaked with ECCs was incubated at 37 °C for 30 min before adding CSSEDF medium. The sponge was cultured in CSSEDF medium for 5 days, and then in N2B27 medium for 8 weeks without any inducing signals.

### Dissociation procedure for human temporomandibular condylar cartilage

The use of primary human tissues was approved by the Ethics Committee of Shanghai Jiaotong University, School of Medicine and written informed consents were signed and acquired from the patients. Human temporomandibular condylar cartilage was harvested from patients who were diagnosed with osteoarthritis or benign condylar hyperplasia and needed condylar excision. The cartilaginous zones were dissected from the mandibular condyle.

### RNA isolation and RNA sequencing

To determine transcriptome profiles over the course of differentiation, five hPSC lines (H1, Hues9, H9, Hues7 and iPSC) were used for biological replicates at various differentiation stages (hPSCs, inducted for 3, 7, 16 and 30 days per cell line; i.e., a total of 25 samples) and were collected for bulk RNA-seq. Total RNA of cells and tissue was extracted using TRIzol reagent (15596026) according to the manufacturer’s protocol. RNA purity and quantification were evaluated using a NanoDrop 2000 spectrophotometer (Thermo Scientific, USA). RNA integrity was assessed using the Agilent 2100 Bioanalyzer (Agilent Technologies, Santa Clara, CA, USA). Then, libraries were constructed using the TruSeq Stranded mRNA LT Sample Prep Kit (Illumina, San Diego, CA, USA) according to the manufacturer’s instructions. Transcriptome sequencing and analysis were conducted by OE Biotech Co., Ltd. (Shanghai, China). The libraries were sequenced on an Illumina HiSeq X Ten platform, and 150 bp paired-end reads were generated. Approximately 50 M raw reads for each sample were generated.

### Differentially expressed gene analysis of bulk RNA-seq data

Raw data (raw reads) were processed using Trimmomatic. The reads containing poly-N and the low-quality reads were removed to obtain clean reads. The clean reads were mapped to the human genome (GRCh38) using HISAT2^62^. The FPKM of each gene was calculated using Cufflinks^63^, and the read counts of each gene were obtained by HTSeq count^64^. Differential expression analysis was performed using the DESeq (2012) R package. An adjusted *P* value [false discovery rate (FDR) suggested by Benjamini and Hochberg] of <0.05 and |log2foldchange| of >1 was set as the threshold for significantly differential expression. Hierarchical cluster analysis of differentially expressed genes (DEGs) was performed to demonstrate the expression pattern of genes in different groups and samples. GO term and KEGG pathway enrichment analyses^65^ of DEGs were performed by using the clusterProfiler and org.Hs.eg.db R packages, respectively, based on the hypergeometric distribution. Gene set variation analysis (GSVA) of DEGs was performed by using the GSVA R package to further analyse the differences between these samples based on the Molecular Signatures Database (MSigDB)^66^. Gene set enrichment analysis (GSEA) of DEGs was performed by using the gseKEGG (organism = “hsa”) and gseGO (ont = “BP”) functions in the clusterProfiler R package^67^. The normalized enrichment score (NES) was calculated, and the threshold for significance was a *P* value of <0.05 and adjusted *P* value (FDR) of <0.25. PCA plots were generated with FPKM values using the function plotPCA in the rgl package of R.

### Single-cell RNA-seq data preprocessing

We acquired single-cell RNA-seq datasets of chondroprogenitors from hiPSCs via the paraxial mesoderm (Cp, NIH Gene Expression Omnibus (GEO) accession number GSM4876130) and chondrogenic differentiation 28 days after the Cp stage (Cp_28D, GSM4876134) from a previously published study^45^. Temporomandibular joint cartilage was acquired from patients with benign condylar hyperplasia. ECCs induced from H1 hPSCs and chondrogenic differentiation 28 days after the ECCs stage (ECC_28D) were collected for single-cell RNA-seq. The Cell Ranger software pipeline (version 5.0.0) provided by 10× Genomics was used to demultiplex cellular barcodes, map reads to the genome and transcriptome using the STAR aligner, and downsample reads as required to generate normalized aggregate data across samples, producing a matrix of gene counts versus cells. We processed the unique molecular identifier (UMI) count matrix using the R package Seurat (version 3.1.1)^68^. To remove low-quality cells and likely multiple captures, which is a major concern in microdroplet-based experiments, we calculated the first quartile (Q1), third quartile (Q3) and interquartile range (IQR) in each sample’s UMI or gene numbers. Cells with UMI or gene numbers less than Q1−1.5*IQR or more than Q3 + 1.5*IQR were filtered out. Following a visual inspection of the distribution of cells by the fraction of mitochondrial genes expressed, we further discarded low-quality cells where >10% of the counts belonged to mitochondrial genes. Additionally, we applied the DoubletFinder package (version 2.0.2) to identify potential doublets^69^. After applying these QC criteria, 12386 and 5123 single cells in TMJ cartilage samples and ECCs were included in the downstream analyses. Library size normalization was performed with the NormalizeData function in Seurat^68^ to obtain the normalized count. Specifically, the global-scaling normalization method “LogNormalize” normalized the gene expression measurements for each cell by the total expression, multiplied by a scaling factor (10,000 by default), and the results were log-transformed.

The top variable genes across single cells were identified using the method described in Macosko et al.^70^. The most variable genes were selected using the FindVariableGenes function (mean.function = FastExpMean, dispersion.function = FastLogVMR) in Seurat^68^. To remove the batch effects in single-cell RNA-sequencing data, FindIntegrationAnchors and IntegrateData functions were used to integrate the data^71^. Graph-based clustering was performed to cluster cells according to their gene expression profile using the FindNeighbours and FindClusters functions in Seurat. Cells were visualized using the UMAP dimensional reduction technique by the RunUMAP function in Seurat. We used the FindAllMarkers function (test.use = bimod) in Seurat to identify marker genes of each cluster. For a given cluster, FindAllMarkers identified positive markers compared with all other cells. Then, we used the R package SingleR^72^, a novel computational method for unbiased cell type recognition of scRNA-seq, with the reference transcriptomic dataset ‘Human Primary Cell Atlas’^73^ to infer the cell of origin of each of the single cells independently and identify cell types. DEGs were identified using the FindMarkers function (test.use = MAST) in Seurat. A *P* value of <0.05 and |log2foldchange| of >0.25 were set as the thresholds for significant differential expression. Then, to further identify the differences between ECCs and Cp, GSEA was performed as mentioned above. For GSEA, the NES was calculated, and a *P* value of <0.05 and an adjusted *P* value (FDR) of <0.25 were considered to be statistically significant. The sequencing and bioinformatics analysis were performed by OE Biotech Co., Ltd. (Shanghai, China).

### Immunostaining and flow cytometry assays

For immunofluorescence assays, ECCs were fixed in 4% paraformaldehyde for 10 min. The fixed cells were washed three times with PBS containing 0.1% Triton X-100 (Sigma-Aldrich) and incubated in a blocking buffer containing 0.1% Triton X-100 and 5% normal donkey serum (Jackson ImmunoResearch Laboratories Inc.) in PBS for 30 mins at room temperature. Tissue samples were fixed in 4% paraformaldehyde, and paraffin-embedded sections were prepared. Serial tissue sections were stained with HE, safranin O (Solarbio, G1371), toluidine blue (Solarbio, G2543) or alcian blue (Solarbio, G1563). For immunohistochemistry, the cells or serial sections were then incubated with primary antibody overnight at 4 °C in a blocking buffer. The cells or serial sections were then stained with the appropriate Alexa Fluor-conjugated secondary antibodies (Invitrogen, 1:1000) in PBS containing 0.1% Triton X-100 for half an hour at room temperature. The primary antibodies are described in Supplementary Table 1.

For indirect flow cytometry, the cells were fixed and permeabilized using a BD Cytofix/Cytoperm kit (BD Biosciences) and then incubated with antibodies against Ki-67 (Abcam, ab15580, 1:200), SOX5 (Abcam, ab94396, 1:200), SOX9 (Abcam, MAB1778, 1:200), and TWIST1 (R&D Systems, AF6230, 1:200) on ice for 1 h and washed three times. The cells were then incubated with Alexa Fluor 488-conjugated AffiniPure donkey anti-rabbit or anti-mouse IgG (H + L) (Jackson ImmunoResearch Laboratories Inc., 1:500) on ice in the dark for 30 mins and washed three times. Flow cytometry analysis was carried out using a FACSCalibur flow cytometer (Becton Dickinson). The data were analysed using FlowJo.

### Quantitative and semiquantitative RT‒PCR

RNA was extracted using the RNeasy Plus Mini Kit in combination with QIAshredder (Qiagen). Reverse transcription was performed with 1 μg RNA using an iScript cDNA Synthesis Kit (Bio-Rad). Semiquantitative PCR was carried out using Platinum PCR SuperMix (Invitrogen). The primers specific for OCT4, NANOG, SOX2, SIX1, TUBB3, ZIC1, KDR, TBX6, PAX1, GATA4, GATA6, SOX7, SP1, SP7, ACAN, OCN, RUNX2, SOX9, SOX5, COL1A, COL2A, ALPL, SPP1, FABP4, CEBPA, APN, β-Actin and GAPDH are listed in Supplementary Table 2. Real-time PCR was carried out using iQ SYBR Green Supermix (Bio-Rad). The expression of genes of interest was normalized to that of GAPDH in all samples. After normalization, the data were transformed as log10 of the target mRNA signal relative to the untreated control sample signal.

### Protein extracts and Western Blotting

Protein lysates were derived from cells using Cell Lysis Buffer (Cell Signalling Technology) containing PMSF (Beyotime Biotechnology) and the concentrations were determined using a Bradford protein assay (Thermo Fisher Scientific). Equivalent amounts of proteins (20 μg) were resolved by sodium dodecyl sulfate–polyacrylamide gel electrophoresis (SDS–PAGE) and then transferred onto NC membranes (Amersham). Membranes were blocked by 5% BSA in Tris-buffered saline and incubated with primary antibodies (dilution in 5% BSA) overnight at 4 °C, then washed and incubated with corresponding second antibodies for 1 h at room temperature. Bands were detected by chemiluminescence reagents (Thermo Fisher Scientific). Primary antibodies can be found in Supplementary Table 1. Secondary antibodies used are the following: anti-mouse IgG, HRP-linked antibody (Cell Signalling Technology, 1:2000) and anti-rabbit IgG, HRP-linked antibody (Cell Signalling Technology, 1:2000). All blots were derived from the same experiment and processed in parallel. The uncropped blots are listed in the Supplementary Figures.

### Scaffold implantation in Nod-SCID mice

Procedures were carried out according to the guidelines of the Ethics Committee of Shanghai Jiaotong University, School of Medicine. Before implantation, ECCs (1 × 10^7^ cells/cm^2^) were seeded on sponges and cultured for 28 days in N2B27 medium. Twelve Nod-SCID mice (6–8 weeks old; Animal Experiment Laboratory of Shanghai Jiao Tong University School of Medicine, Shanghai, China) were anaesthetised with isoflurane. Cell-seeded or empty scaffolds were subcutaneously transplanted onto the dorsum of each animal. The incision was closed with sterile sutures. Transplants were harvested after 4 weeks for histological analysis.

### Rat cartilage defect repair

Twelve 5- to 6-week-old female Sprague‒Dawley rats (provided by the Animal Experimental Laboratory of Shanghai Jiao Tong University School of Medicine) were used in this study. The rats were housed under specific pathogen-free conditions. GFP-labelled ECCs (2 × 10^7^ cells/cm^2^) were seeded on sponges and cultured for 28 days in N2B27 medium. The rats were anesthetized with 2.5% isoflurane. The anterior articular surface of the distal femur was exposed, and a 1 mm-diameter full-thickness cylindrical cartilage defect (1 mm deep) was made using an electric trephine on the articular surface of the femoral patellar groove. Sponges with or without GFP-labelled ECCs were implanted into the right or left knee defects, respectively. The immunosuppressant cyclosporine A (NO. C-6000, USA) dissolved in PBS (625 mg/1000 mL) was intraperitoneally injected every day. Regenerated tissue samples were collected 4 weeks later and rinsed with PBS. After evaluating GFP expression under a fluorescence stereoscope (SMZ18, Nikon, Japan), the harvested tissues were fixed in 4% paraformaldehyde, dehydrated and embedded in paraffin for histological analysis.

### Reporting summary

Further information on research design is available in the Nature Research Reporting Summary linked to this article.

## Supplementary information

Supplementary Material

Reporting Summary

## Data Availability

The datasets generated during and/or analysed during the current study are available from the corresponding author upon reasonable request. RNA-sequencing data and single-cell RNA-seq data have been deposited in NCBI Sequence Read Archive (SRA) under the accession code HRA003076, HRA003116, and HRA003024.
