# Supplementary material for: Rapid induction and long-term self-renewal of neural crest-derived ectodermal chondrogenic cells from hPSCs
Source: NPJ Regen Med. 2022 Dec 8;7:69. doi: 10.1038/s41536-022-00265-0 (PMC9729200; doi:10.1038/s41536-022-00265-0)
Supplement: Supplementary file 1 — Supplementary Material [file 41536_2022_265_MOESM1_ESM.pdf]

Supplementary figures and tables

This section contains eleven supplementary figures (1-11), full blots and corresponding molecular weight marker images of the blot strips displayed in figures, and 2 supplementary tables detailing the reagents and primer sequence used in this study.

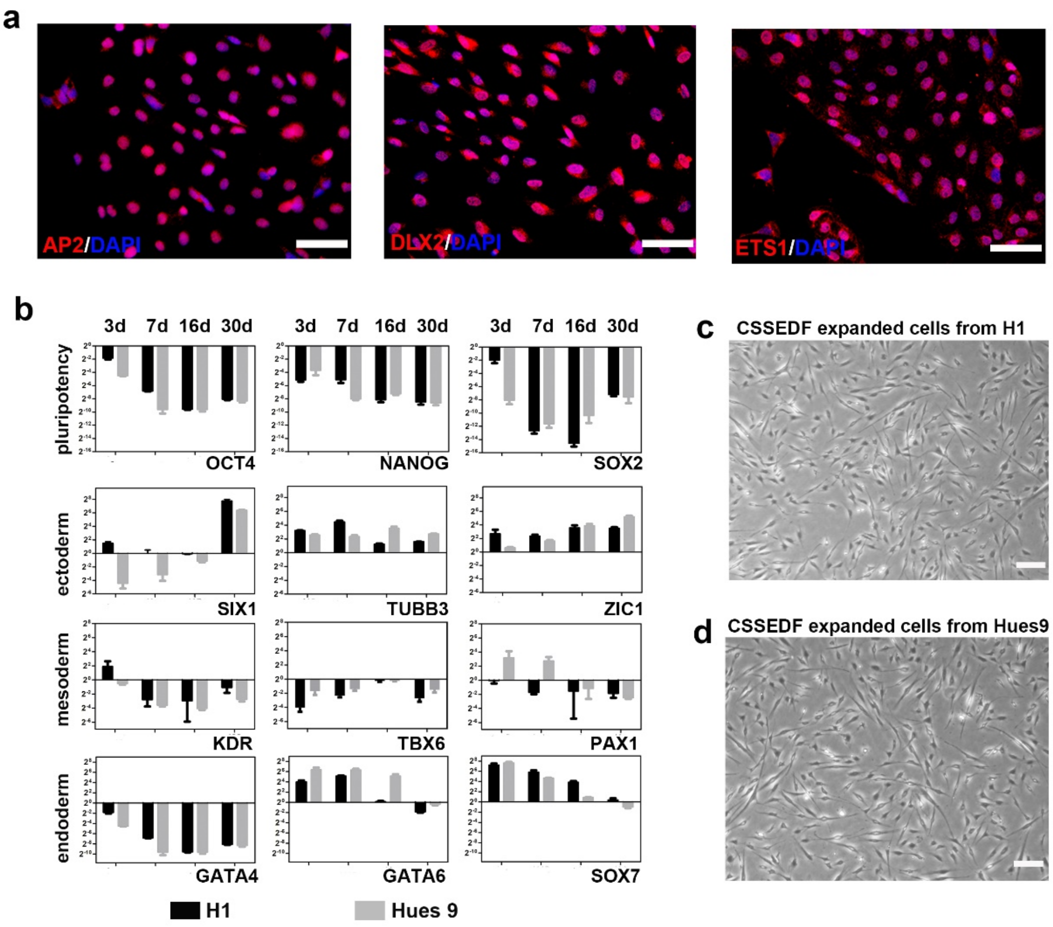

Supplementary Figure 1. Changes of morphology and triploblastic markers during induction. **a.** Immunocytochemistry showing that hPSCs treated with BMP4 and SB431542 for 7 d were positive for the NC genes AP2, DLX2 and ETS1. Scale bars, 50 μm. **b.** Gene expressions of pluripotency markers OCT4, NANOG and SOX2, ectodermal markers SIX1, TUBB3, and ZIC1, mesodermal markers KDR, TBXT and

PAX1 and endodermal markers GATA4, GATA6 and SOX7 at different induction points were analysed by real-time PCR. **c.d.** Morphology of CSSEDF-expanded cells from H1 and Hues9 after induction. Scale bars, 50 μm.

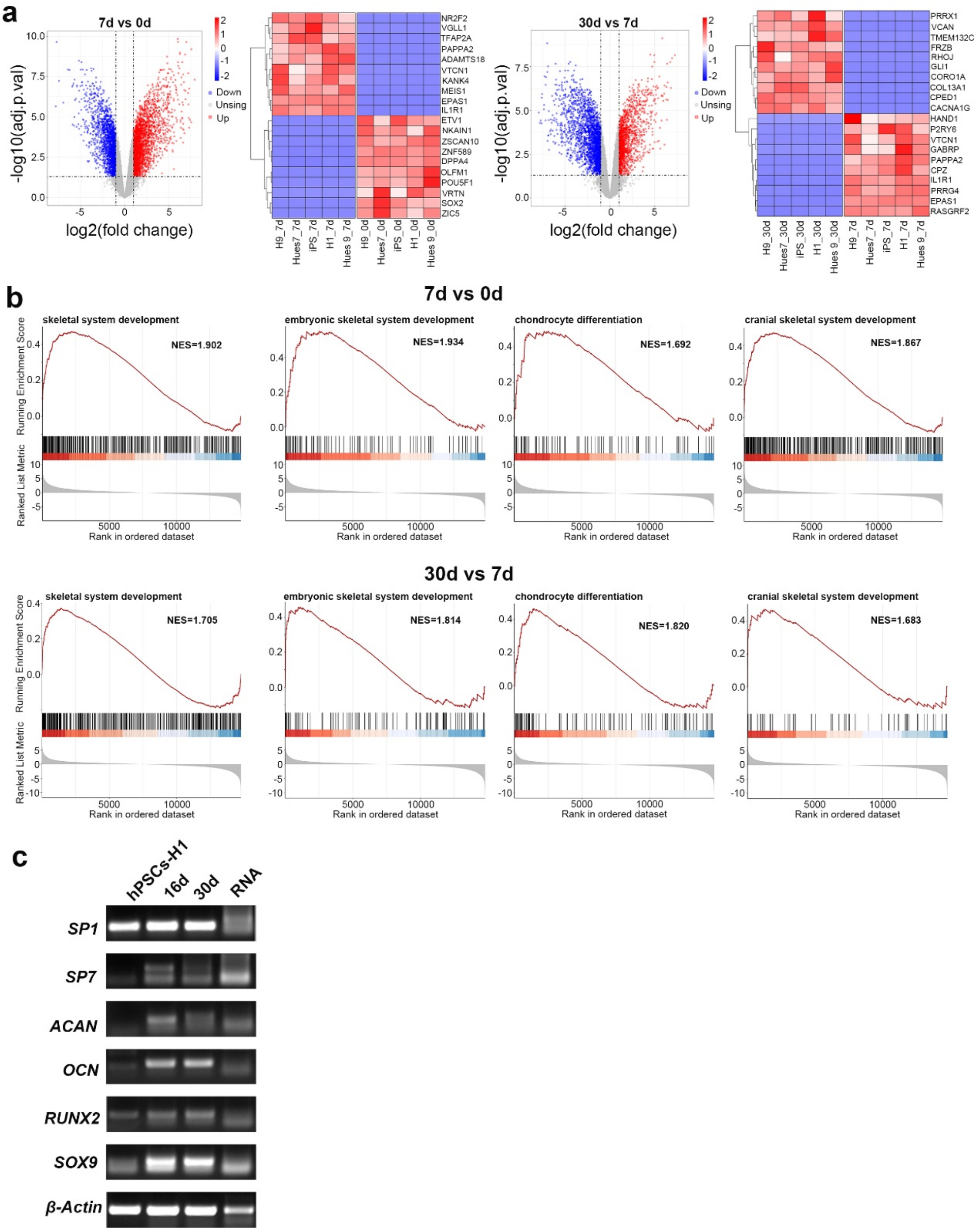

Supplementary Figure 2. DEGs reveal chondrogenic differentiation of five hPSC lines by bulk RNA-seq. **a.** The top 10 upregulated and downregulated genes at 7 d and 30 d. **b.** Gene set enrichment analysis showing a significant upregulation of the cranial skeleton and cartilage gene set during induction. **c.** PCR showing that chondrogenic genes are up-regulated during and after induction.

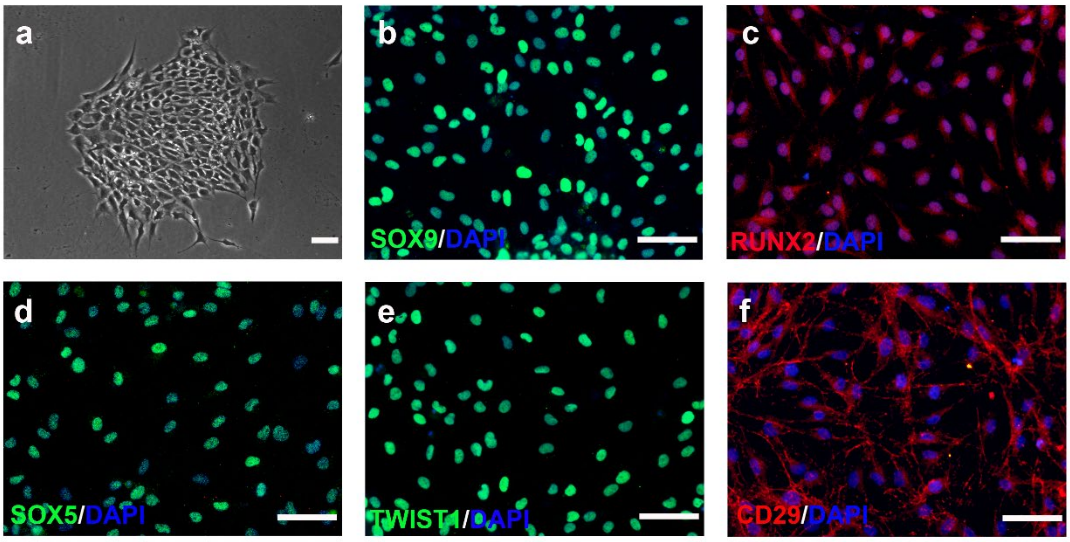

Supplementary Figure 3. CSSEDF-expanded cells at late-passage stably self-renew and share markers representing CPCs/CSCs. **a**. A single CSSEDF-expanded cell (passage 16) colony on a Matrigel-coated surface. Scale bars, 50 μm. **b-f.** Immunocytochemistry showing that CSSEDF-expanded cells (passage 18) expressed genes identified as CPC/CSC markers, including SOX9, RUNX2, SOX5, TWIST1 and CD29. Scale bars, 50 μm.

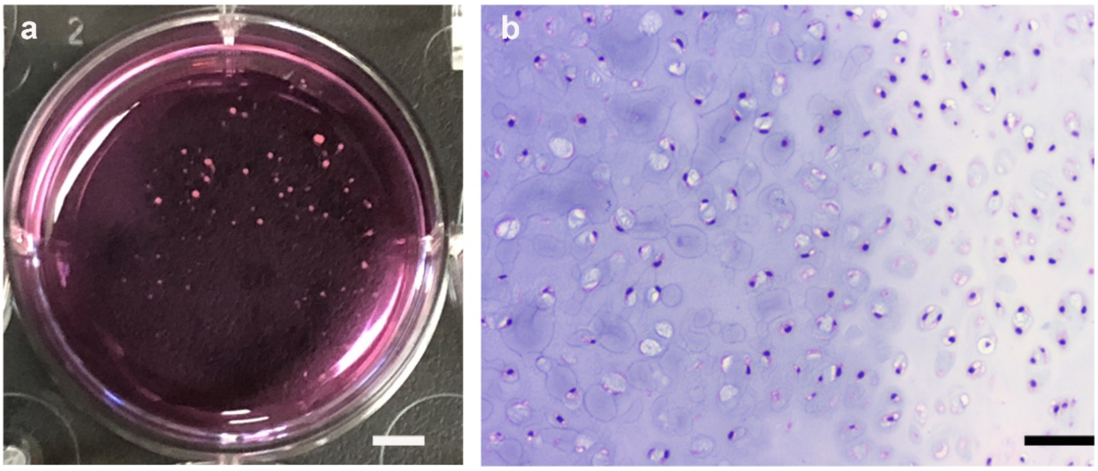

Supplementary Figure 4. Spontaneous differentiation of CSSEDF-expanded cells cultured in N2B27 basal medium in a monolayer. **a.** Gross appearance showing that scattered white flakes were visible in the petri dish. Scale bars, 1 mm. **b.** Cartilage lacuna was formed and the ECM was rich in monolayer culture. Scale bars, 50 μm.

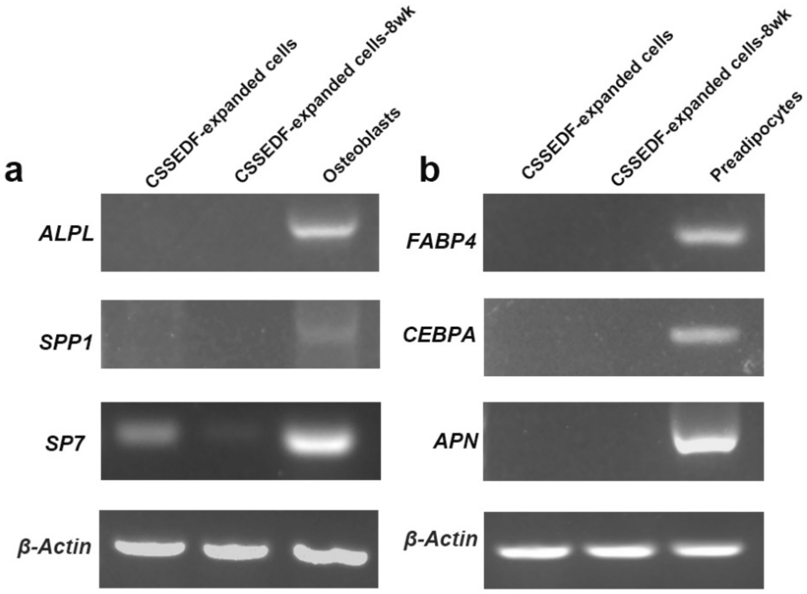

Supplementary Figure 5. Gene expression for osteogenic and adipogenic differentiation of CSSEDF-expanded cells cultured with N2B27 medium for 8 weeks. **a.** The expression of osteogenic markers, ALPL, SPP1, and SP7 was virtually

undetectable. **b.** The expression of adipogenic markers, FABP4, CEBPA and APN was virtually undetectable.

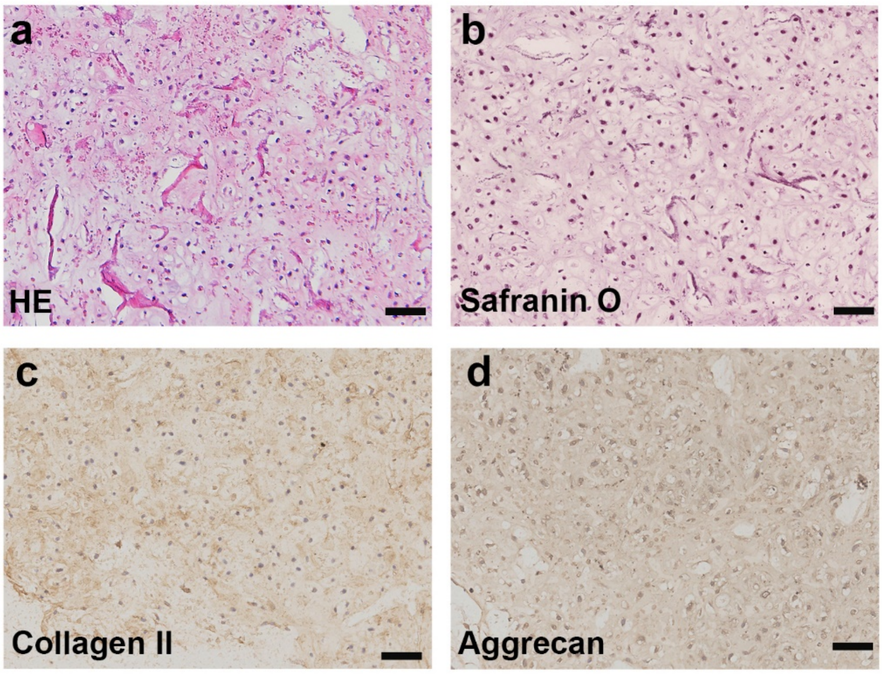

Supplementary Figure 6. Histological staining of ECCs in sponge cultured with N2B27 medium for 4 weeks. **a.** The cells in sponge were largely immature chondrocytes. **b.** The chondrocytes were week positive for safranin O staining. **c-d.** Immunocytochemistry showing that collagen II and aggrecan were positive in the chondrocytes. Scale bars, 50 μm.

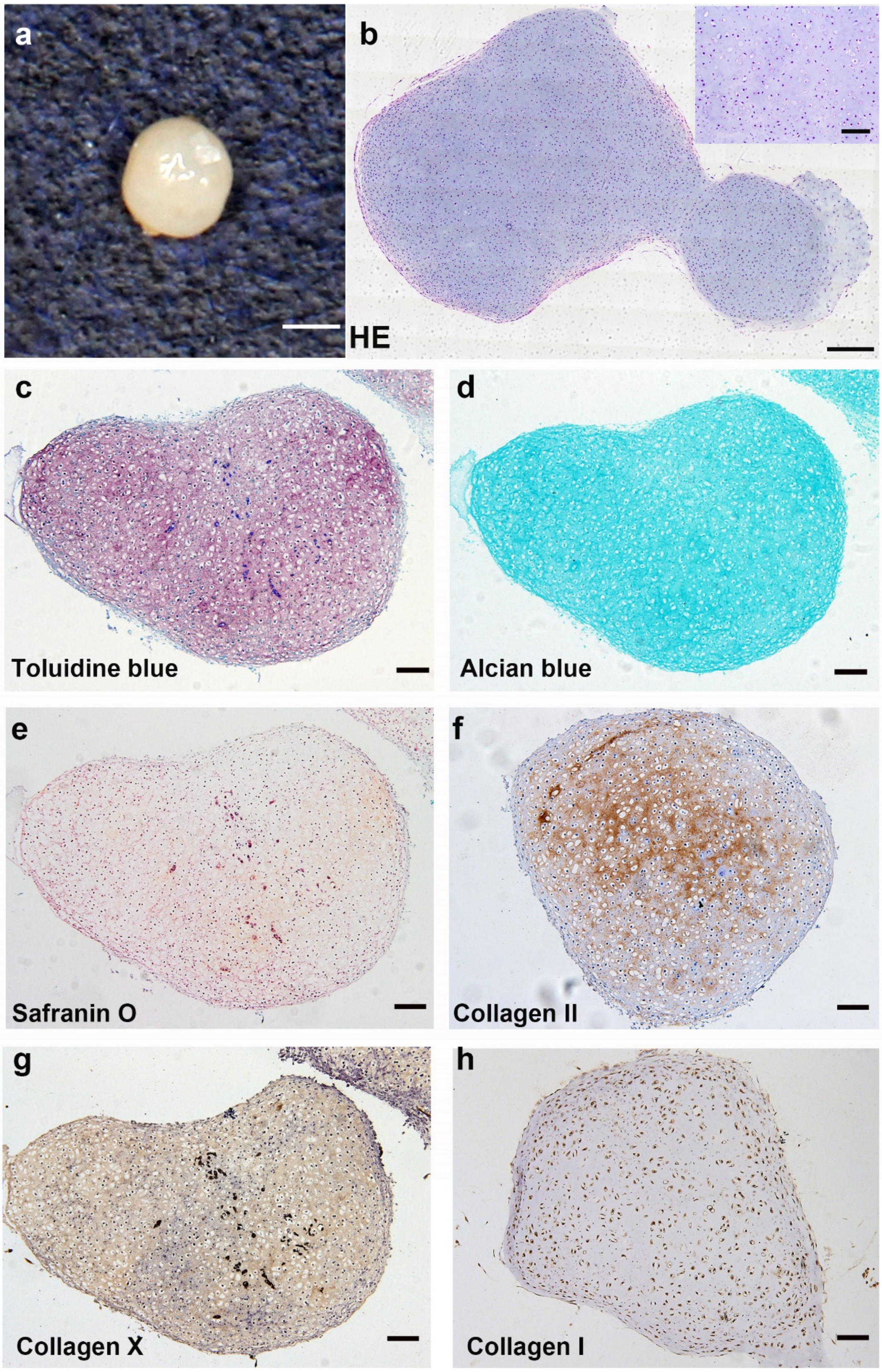

Supplementary Figure 7. Spontaneous differentiation of ECCs cultured in N2B27 basal medium in suspension. **a.** Gross appearance of ECCs seeded in suspension for 8

weeks. Scale bars, 1 mm. **b.** Panoramic scanning showing the formation of cartilage masses (inset: HE staining enlargement). Scale bars, 200 μm. (Inset) Scale bars, 50 μm. **c-e.** The chondrocytes were positive for toluidine blue, alcian blue and week positive for safranin O staining. Scale bars, 100 μm. **f-k.** The chondrocytes express common chondrocytic markers, including collagen II, collagen X, and collagen I. Scale bars, 100 μm.

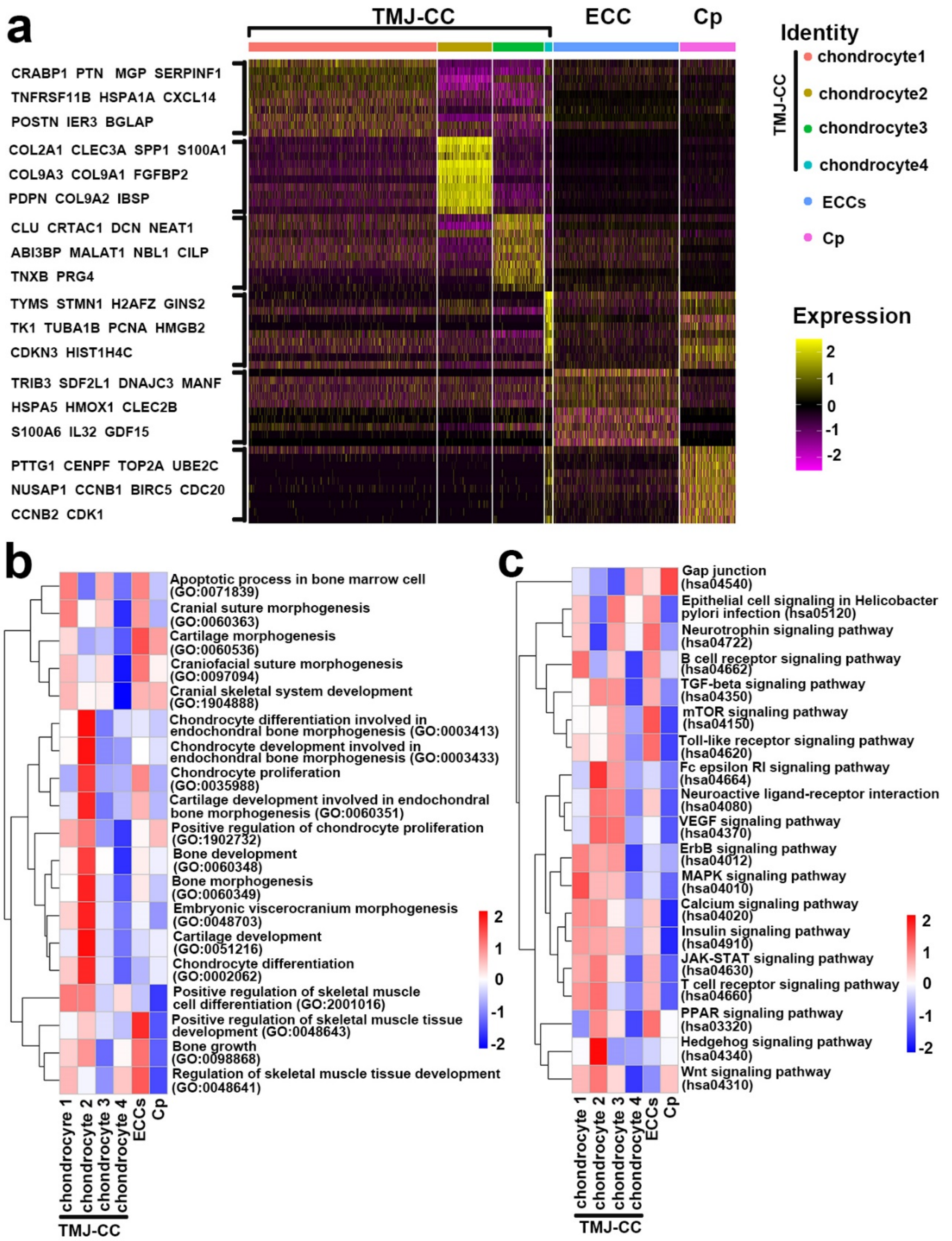

Supplementary Figure 8. Comparison among TMJ-CC, ECCs and Cp by single-cell transcriptomics. **a.** The top 10 SDE genes in each cell type in TMJ-CC, ECCs and Cp. **b.** GO analysis of TMJ-CC, ECCs and Cp. **c.** KEGG analyses of different cell types in TMJ-CC, ECCs and Cp.

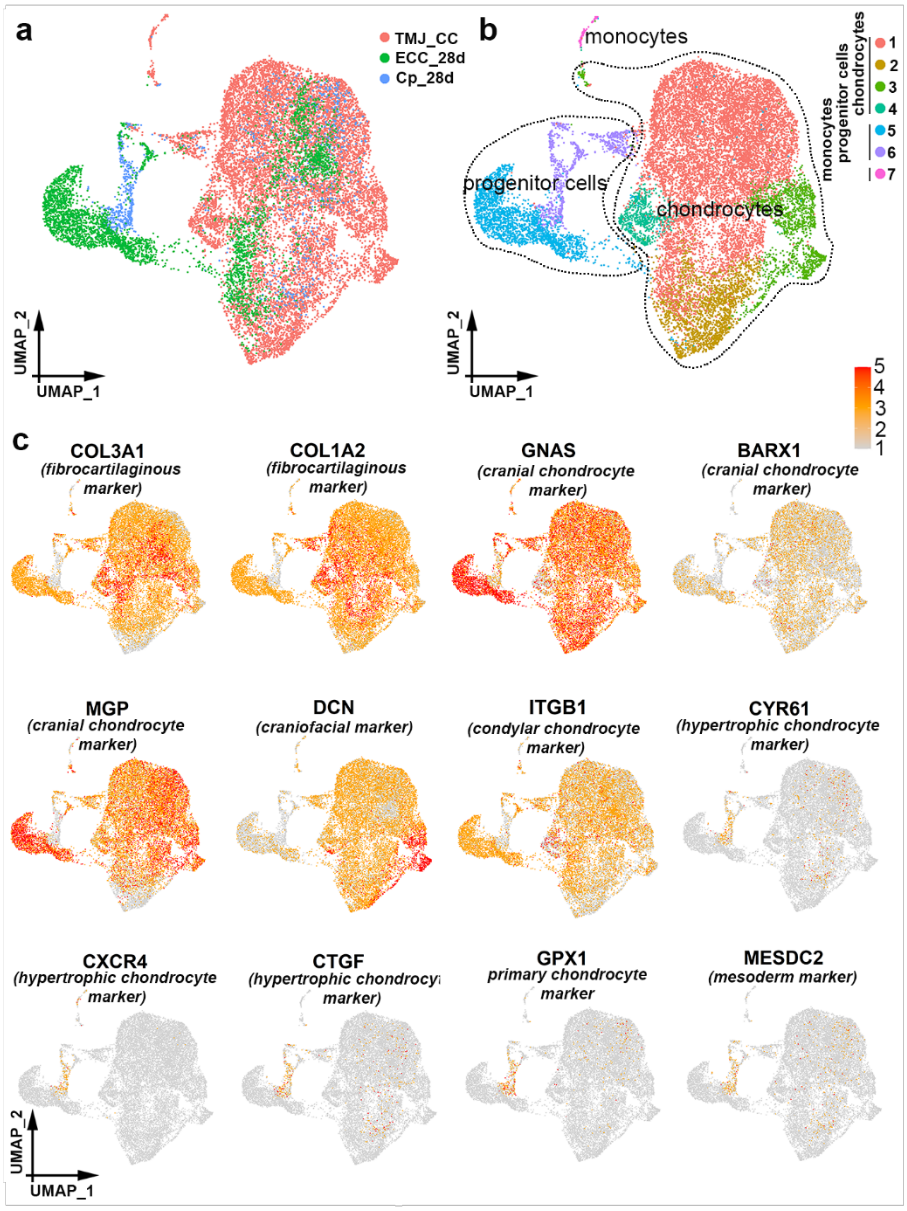

Supplementary Figure 9. Single-cell transcriptome analysis of TMJ-CC and the cells differentiated 28 days from ECCs and Cp. **a.** UMAP showing a cluster of cells in ECC-28d overlapped with the TMJ-CC. **b.** Dimension-reduction presentation of combined single-cell transcriptome data from (**a**), labeled with corresponding cell categories and colored according to its cell type identity. **c.** Expression patterns of selected markers projected on the UMAP plot (**a**).

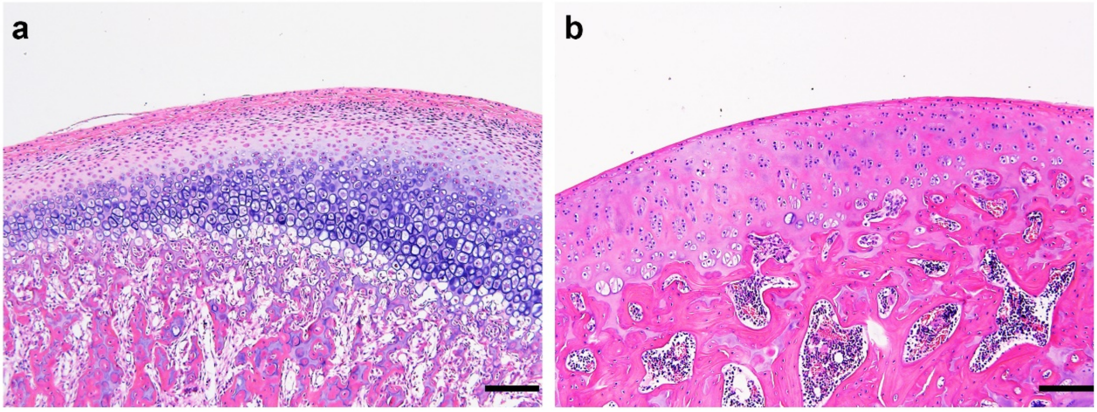

Supplementary Figure 10. HE staining demonstrated histological differences in TMJ condylar cartilage **(a)** and knee cartilage **(b)**. Scale bar, 100μm.

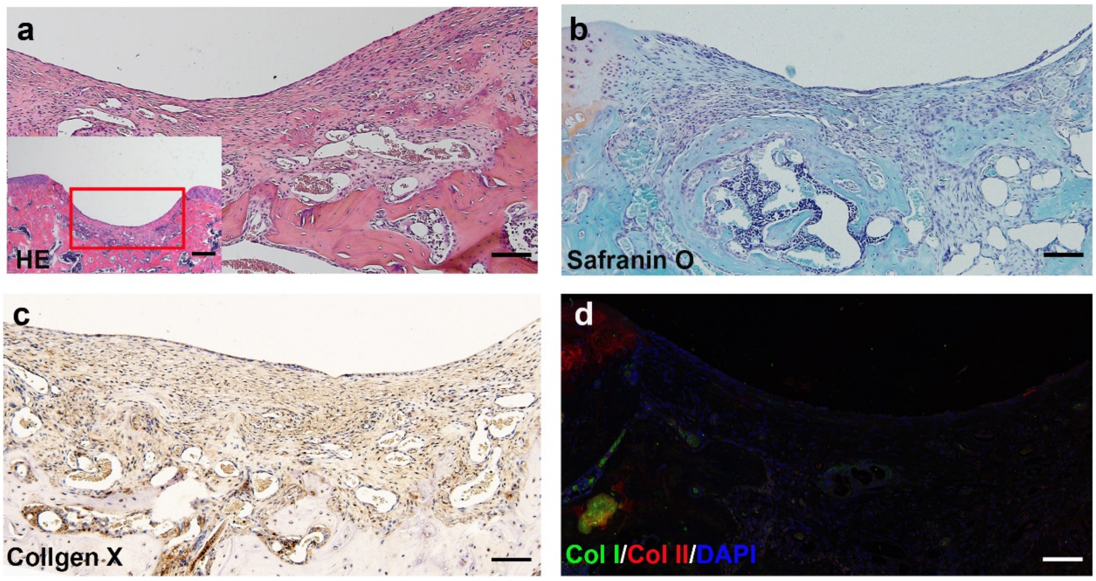

Supplementary Figure 11. Histological staining of the tissue implanted with a control collagen sponge. **a.** The scar tissue was thinner and consisted of fibrocytes with vascular invasion in the control group. **b.** The scar tissue was negative for safranin O staining. **c.** Collagen X expressed in the scar tissue. **d.** immunofluorescence costaining showing the absence of collagen I and collagen II in the control group. Scale bar, 100μm.

Full blot image and corresponding molecular weight marker of blot strips shown in Figure 7b

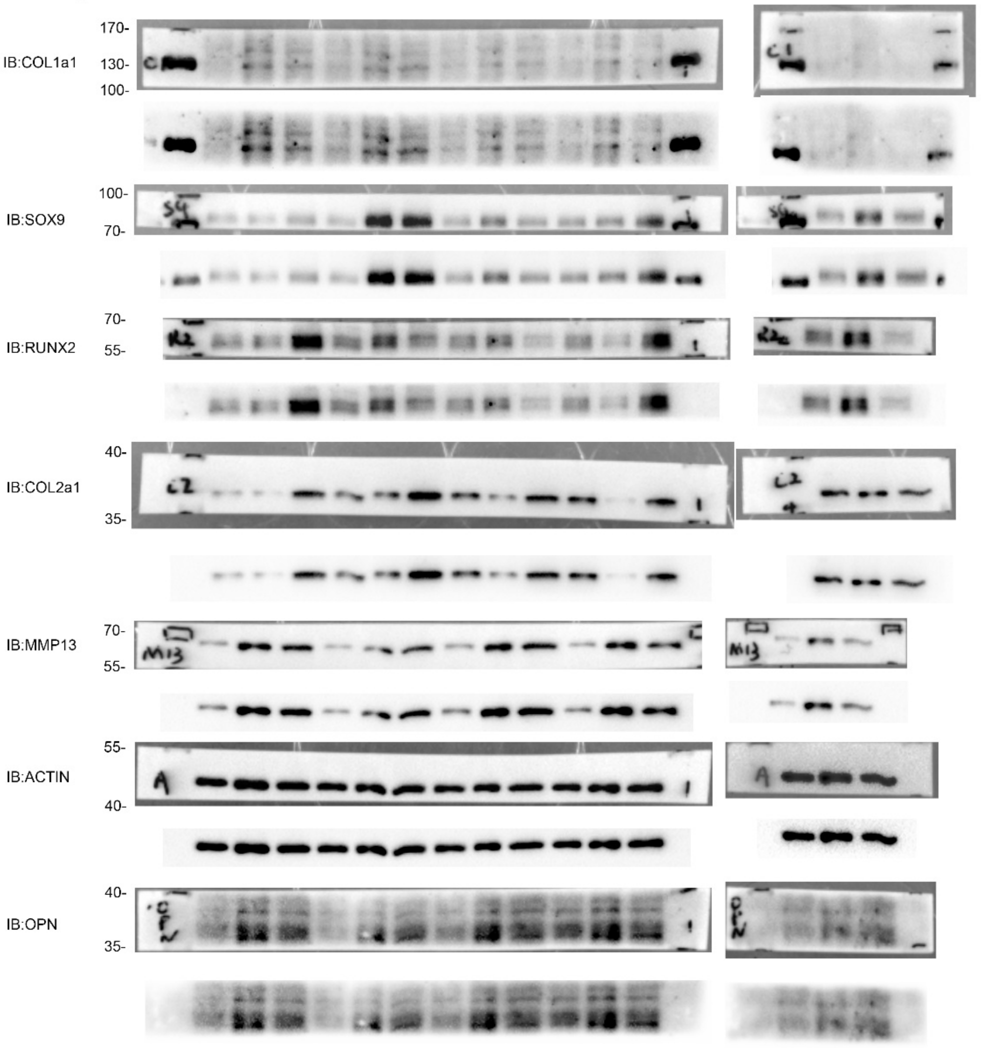

Full blot image and corresponding molecular weight marker of blot strips shown in Figure 7c

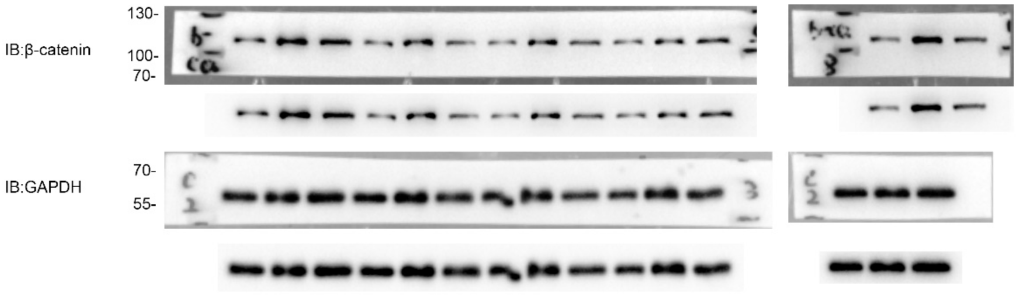

Full blot image and corresponding molecular weight marker of blot strips shown in Figure 7d

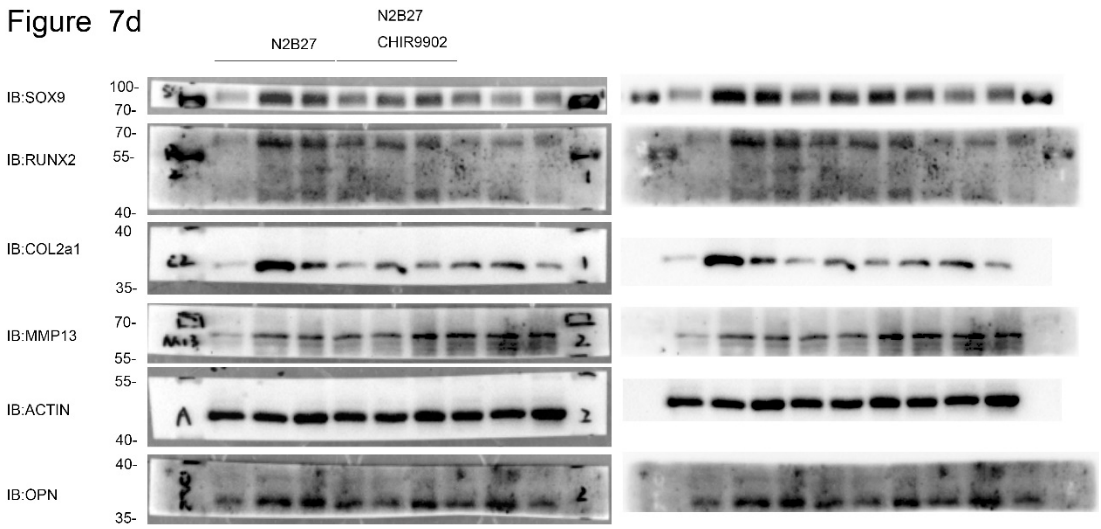

Supplementary Table 1. Antibodies used in immunocytochemistry

antibodies	source	dilution
AP2	Santa, sc12726	200
DLX2	Millipore, AB5726	500
ETS1	Santa, sc55581	500
SIX1	SIGMA, HPA001893	1000
Nestin	R&, MAB1259	500
SOX5	Abcam, ab94396	200
CD29	R&D, MAB1778	200
SOX9	Millipore, AB5535	200
RUNX2	R&D, MAB2006	200
TWIST1	R&D, AF6230	500
FOXC2	R&D, AF5044	500
FOXC1	Abcam, ab227977	500
MSX1	R&D, AF5045	500
Collagen II	Millipore, MAB8887	200
Collagen I	Affinity, AF7001	200
Collagen X	BIOSS, bs-0554R	200
Aggrecan	Abcam, ab3778	100

Lubricin	BIOSS, bs-11175R	200
Ki67	Abcam, ab15580	500
MS X HU Nuclei	R&D, MAB1281	100
Rb pAb to GFP	Abcam, ab290	100
toluidine blue	Solarbio, G2543	
alcian blue	Solarbio, G1563	
safranin O	Solarbio, G1371	

Supplementary Table 2. PCR primers used in real-time PCR experiments

Gene name	Forward (5'-3')	Reverse (5'-3')
OCT4	GATGTGGTCCGAGTGTGGTTCTG	CGAGGAGTACAGTGCAGTGAAGTG
NANOG	AGCAATGGTGTGACGCAGAAGG	ACCAGGTCTGAGTGTTCCAGGAG
SOX2	GGATAAGTACACGCTGCCCG	ATGTGCGCGTAACTGTCCAT
SIX1	CTGCCGTCGTTTGGCTTTAC	GCTCTCGTTCTTGTGCAGGT
TUBB3	GACTCCCTTGAACAGGGACAG	TTGTTCTCAAGAGATGCCCC
ZIC1	CACGCGGGACTTTCTGTTC	TGCCCGTTGACCACGTTAG
KDR	GTGATCGGAAATGACACTGGAG	CATGTTGGTCACTAACAGAAGCA
TBX6	CATCCACGAGAATTGTACCCG	AGCAATCCAGTTTAGGGGTGT
PAX1	AAGTACAATGTGCCTTCGGTGAGC	CGACGGCGGCTGCTTACTTG
GATA4	GCGGAAAGAGGGGATCCAAA	CTTGTGGGGAGAGCTTCAGG
GATA6	TGCCAACTGTCACACCACAA	CATAGCAAGTGGTCTGGGCA
SOX7	AGCTGTCGGATGGACAATCG	CCACGACTTTCCCAGCATCT
SOX9	CAGACGCACATCTCCCCCAA	CTCTCGCTTCAGGTCAGCCTT
COL1A1	GGACACAGAGGTTTCAGTGGT	GCACCATCATTTCCACGAGC
SOX5	CTCCAGCAACAGATCCAGGTT	AGTTGGGATCAGCTGAACAGG
COL2A1	GGTGAGCCATGATTCGCCTC	GACAGTCCCAGTGTCACAGAC
OCN	CCTCACACTCCTCGCCCTAT	GGGTCTCTTCACTACCTCGC
RUNX2	GCGCATTCCTCATCCCAGTA	GGCTCAGGTAGGAGGGGTAA
ACAN	ACTCTGGGTTTTCGTGACTCT	ACACTCAGCGAGTTGTCATGG
SP1	TGGCAGCAGTACCAATGGC	CCAGGTAGTCCTGTCAGAACTT
SP7	CCTCTGCGGGACTCAACAAC	AGCCCATTAGTGCTTGTAAAGG

ALPL	AGTGCTCTGCGCAGGATTG	TGGAGACACCCATCCCATCT
SPP1	AGCAGAATCTCCTAGCCCCA	ACGGCTGTCCCAATCAGAAG
FABP4	ATGGGGGTGTCCTGGTACAT	AACGTCCCTTGGCTTATGCT
APN	ACTGCAGTCTGTGGTTCTGA	GCACCTTCTCCAGGTTCTCC
CEBPA	TACAGCAGACCCCCATGAGA	CCCACGACCTAGCTTTCTGG
