# Supplementary material for: Rapid induction and long-term self-renewal of neural crest-derived ectodermal chondrogenic cells from hPSCs
Source: NPJ Regen Med. 2022 Dec 8;7:69. doi: 10.1038/s41536-022-00265-0 (PMC9729200; doi:10.1038/s41536-022-00265-0)
Supplement: Supplementary file 2 — Reporting Summary [file 41536_2022_265_MOESM2_ESM.pdf]

nature portfolio

Corresponding author(s): Wenlin Li

Last updated by author(s): Sep 11, 2022

Reporting Summary

Nature Portfolio wishes to improve the reproducibility of the work that we publish. This form provides structure for consistency and transparency in reporting. For further information on Nature Portfolio policies, see our Editorial Policies and the Editorial Policy Checklist.

Statistics

For all statistical analyses, confirm that the following items are present in the figure legend, table legend, main text, or Methods section.

n/a	Confirmed	
☐	☒	The exact sample size (*n*) for each experimental group/condition, given as a discrete number and unit of measurement
☒	☐	A statement on whether measurements were taken from distinct samples or whether the same sample was measured repeatedly
☒	☐	The statistical test(s) used AND whether they are one- or two-sided<br>*Only common tests should be described solely by name; describe more complex techniques in the Methods section.*
☒	☐	A description of all covariates tested
☒	☐	A description of any assumptions or corrections, such as tests of normality and adjustment for multiple comparisons
☒	☐	A full description of the statistical parameters including central tendency (e.g. means) or other basic estimates (e.g. regression coefficient) AND variation (e.g. standard deviation) or associated estimates of uncertainty (e.g. confidence intervals)
☒	☐	For null hypothesis testing, the test statistic (e.g. *F*, *t*, *r*) with confidence intervals, effect sizes, degrees of freedom and *P* value noted<br>*Give P values as exact values whenever suitable.*
☒	☐	For Bayesian analysis, information on the choice of priors and Markov chain Monte Carlo settings
☒	☐	For hierarchical and complex designs, identification of the appropriate level for tests and full reporting of outcomes
☒	☐	Estimates of effect sizes (e.g. Cohen's *d*, Pearson's *r*), indicating how they were calculated

Our web collection on statistics for biologists contains articles on many of the points above.

Software and code

Policy information about availability of computer code

Data collection	We acquired single-cell RNA-seq datasets of chondroprogenitors from hiPSCs(Cp, NIH Gene Expression Omnibus (GEO) accession number GSM4876130) and chondrogenic differentiation 28 days after the Cp stage (Cp_28D, GSM4876134) by wget (version 1.9.1).
Data analysis	The Cell Ranger software pipeline (version 5.0.0) provided by 10× Genomics was used to demultiplex cellular barcodes, map reads to the genome and transcriptome using the STAR aligner(version 2.7.9a), and downsample reads as required to generate normalized aggregate data across samples, producing a matrix of gene counts versus cells. We processed the unique molecular identifier (UMI) count matrix using the R package Seurat (version 3.1.1).

For manuscripts utilizing custom algorithms or software that are central to the research but not yet described in published literature, software must be made available to editors and reviewers. We strongly encourage code deposition in a community repository (e.g. GitHub). See the Nature Portfolio guidelines for submitting code & software for further information.

Data

Policy information about availability of data

All manuscripts must include a data availability statement. This statement should provide the following information, where applicable:

- Accession codes, unique identifiers, or web links for publicly available datasets
- A description of any restrictions on data availability
- For clinical datasets or third party data, please ensure that the statement adheres to our policy

Provide your data availability statement here.

Human research participants

Policy information about studies involving human research participants and Sex and Gender in Research.

Reporting on sex and gender	Two human temporomandibular joint (TMJ) tissue samples were used in the present study. As TMJ diseases usually affect females, the patients' TMJ samples in the this study were both from Chinese females. The use of primary human tissues was approved by the Ethics Committee of Shanghai Jiaotong University, School of Medicine and written informed consents were signed and acquired from the patients.
Population characteristics	See above.
Recruitment	Patients who were diagnosed with osteoarthritis or benign condylar hyperplasia and needed condylar excision were recruited in the present study.
Ethics oversight	The use of primary human tissues was approved by the Ethics Committee of Shanghai Jiaotong University, School of Medicine.

Note that full information on the approval of the study protocol must also be provided in the manuscript.

Field-specific reporting

Please select the one below that is the best fit for your research. If you are not sure, read the appropriate sections before making your selection.

☒ Life sciences ☐ Behavioural & social sciences ☐ Ecological, evolutionary & environmental sciences

For a reference copy of the document with all sections, see nature.com/documents/nr-reporting-summary-flat.pdf

Life sciences study design

All studies must disclose on these points even when the disclosure is negative.

Sample size	No sample-size calculation was performed. No statistical method was used to determine sample size. Sample size was chosen based on previous experience and standards in the field.
Data exclusions	No data were excluded from the analyses.
Replication	The reproducibility of the induction process was peformed in five hPSCs.<br>All PCR, WB, flow cytometry, IHC, and IHF experiments were done in triplicate.<br>All attempts of replication were successful and gave similar results
Randomization	Randomization was not relevant to the present study, as we measured physical values in the same condition , i.e. the value of RT-PCR, which is not influenced by the observer.
Blinding	Blinding was not relevant to the present study. The persons performing sample preparation and experiments were unaware of the sample identity. All localization data have been documented and are available upon reasonable request.

Reporting for specific materials, systems and methods

We require information from authors about some types of materials, experimental systems and methods used in many studies. Here, indicate whether each material, system or method listed is relevant to your study. If you are not sure if a list item applies to your research, read the appropriate section before selecting a response.

Materials & experimental systems

n/a	Involved in the study	
☐	☒	Antibodies
☐	☒	Eukaryotic cell lines
☒	☐	Palaeontology and archaeology
☐	☒	Animals and other organisms
☒	☐	Clinical data
☒	☐	Dual use research of concern

Methods

n/a	Involved in the study	
☒	☐	ChIP-seq
☐	☒	Flow cytometry
☒	☐	MRI-based neuroimaging

Antibodies

Antibodies used	Detailed information about the antibodies used in this study are provided in Supplementary Table 1.
Validation	In addition to the information provided in Supplementary Table 1, validation of each primary antibody can be acquired from the manufacturer's website.

Eukaryotic cell lines

Policy information about cell lines and Sex and Gender in Research

Cell line source(s)	H1 (human, WiCell, WA01)<br>Hues9(human, Harvard University)<br>H9 (human, , WiCell, WA09)<br>Hues7(human, Harvard University)<br>iPSC(human, ATCC-DYR0100)<br>preadipocytes(human, Shanghai Cell Bank BFN608007090)<br>osteoblasts(human, Shanghai Cell Bank hFOB1.19)
Authentication	None of the cell lines have been authenticated.
Mycoplasma contamination	All these cells tested negative for mycoplasma contamination on a routine base.
Commonly misidentified lines<br>(See ICLAC register)	No commonly misidentified cell lines were used.

Animals and other research organisms

Policy information about studies involving animals; ARRIVE guidelines recommended for reporting animal research, and Sex and Gender in Research

Laboratory animals	No commonly misidentified cell lines were used.
Wild animals	This study didn't involve wild animals.
Reporting on sex	Female Sprague–Dawley rats were used in this study. As temporomandibular joint diseases usually affect female patients, we selected female SD rats in the cartilage defect model.<br>Nod-SCID mice which were used in scaffold implantation comprised of 6 female mice and 6 male mice.
Field-collected samples	This study didn't involve sample collected from the field.
Ethics oversight	Animal experiments were approved by Ethics Committee of Shanghai Jiaotong University, School of Medicine.

Note that full information on the approval of the study protocol must also be provided in the manuscript.

Flow Cytometry

Plots

Confirm that:

☒ The axis labels state the marker and fluorochrome used (e.g. CD4-FITC).

☒ The axis scales are clearly visible. Include numbers along axes only for bottom left plot of group (a 'group' is an analysis of identical markers).

☒ All plots are contour plots with outliers or pseudocolor plots.

☒ A numerical value for number of cells or percentage (with statistics) is provided.

Methodology

Sample preparation	For indirect flow cytometry, the cells were fixed and permeabilized using a BD Cytofix/Cytoperm kit (BD Biosciences) and then incubated with antibodies against Ki-67 (Abcam, ab15580, 1:200), SOX5 (Abcam, ab94396, 1:200), SOX9 (Abcam, MAB1778, 1:200), and TWIST1 (R&D Systems, AF6230, 1:200) on ice for 1 h and washed three times. The cells were then incubated with Alexa Fluor 488-conjugated AffiniPure donkey anti-rabbit or anti-mouse IgG (H+L) (Jackson ImmunoResearch Laboratories Inc., 1:500) on ice in the dark for 30 min and washed three times. Flow cytometry analysis was carried out using a FACSCalibur flow cytometer (Becton Dickinson).
Instrument	Shanghai Ninth People's Hospital, Shanghai Jiao Tong University School of Medicine
Software	FlowJo (10.0.7) were used to analyze flow cytometry.
Cell population abundance	The purity of the samples was provided in Fig 2m. Target cells are labeled with fluorescent markers and analyzed using FlowJo after gating out cellular debris.
Gating strategy	Forward and side scatter gating was used to identify the cells of interest.

☒ Tick this box to confirm that a figure exemplifying the gating strategy is provided in the Supplementary Information.
